# Green Synthesis of Metal Oxides Semiconductors for Gas Sensing Applications

**DOI:** 10.3390/s22134669

**Published:** 2022-06-21

**Authors:** Mehran Dadkhah, Jean-Marc Tulliani

**Affiliations:** Lince Laboratory, INSTM R.U., Department of Applied Science and Technology, Politecnico di Torino, Corso Duca Degli Abruzzi 24, 10129 Torino, Italy; mehran.dadkhah@polito.it

**Keywords:** biological synthesis, green chemistry, metal oxide nanoparticles, metallic nanoparticles, gas sensors

## Abstract

During recent decades, metal oxide semiconductors (MOS) have sparked more attention in various applications and industries due to their excellent sensing characteristics, thermal stability, abundance, and ease of synthesis. They are reliable and accurate for measuring and monitoring environmentally important toxic gases, such as NO_2_, NO, N_2_O, H_2_S, CO, NH_3_, CH_4_, SO_2_, and CO_2_. Compared to other sensing technologies, MOS sensors are lightweight, relatively inexpensive, robust, and have high material sensitivity with fast response times. Green nanotechnology is a developing branch of nanotechnology and aims to decrease the negative effects of the production and application of nanomaterials. For this purpose, organic solvents and chemical reagents are not used to prepare metal nanoparticles. On the contrary, the synthesis of metal or metal oxide nanoparticles is done by microorganisms, either from plant extracts or fungi, yeast, algae, and bacteria. Thus, this review aims at illustrating the possible green synthesis of different metal oxides such as ZnO, TiO_2_, CeO_2_, SnO_2_, In_2_O_3_, CuO, NiO, WO_3,_ and Fe_3_O_4_, as well as metallic nanoparticles doping.

## 1. Introduction

In recent years, the population growth and rapid expansion of industries, especially in textiles, leather, steel, and dyes manufacturing, has led to noticeable negative effects on the environment and health implications [1,2]. These challenges require environmentally sustainable policies and solutions for modification, reduction, and prevention of emissions. Therefore, the demand for alternative ways to synthesize nanoparticles has drawn significant interest worldwide, which has caused the development of green nanobiotechnology or the green nanotechnology concept. These biological methods are eco-friendly with a minimum impact on human health, living organisms, and the environment [3].

Green chemistry is based on 12 principles [4,5] that include:

- Reducing waste/by-product.
- Synthesis of chemicals and products with higher safety.
- Design of chemical synthesis with lower toxicity.
- Utilization of renewable and environmentally precursors.
- Use of effective catalysts.
- Reduce essential derivatization.
- Production of green products with the maximum proportion of raw materials (atom economy).
- Control and decrement of pollution using less toxic chemicals.
- Increase of energy efficiency by the synthesis at room temperature with low pressure.
- Usage of solvents and reaction conditions with a higher level of safety, such as water, ethanol, acetone, and methanol.
- Design to degradation of unrecycled materials at the end of the useful life.
- Choose the suitable substance for the chemical process to minimize the possibility of accidents such as explosions, fires, etc. [6].

**Figure 1:**
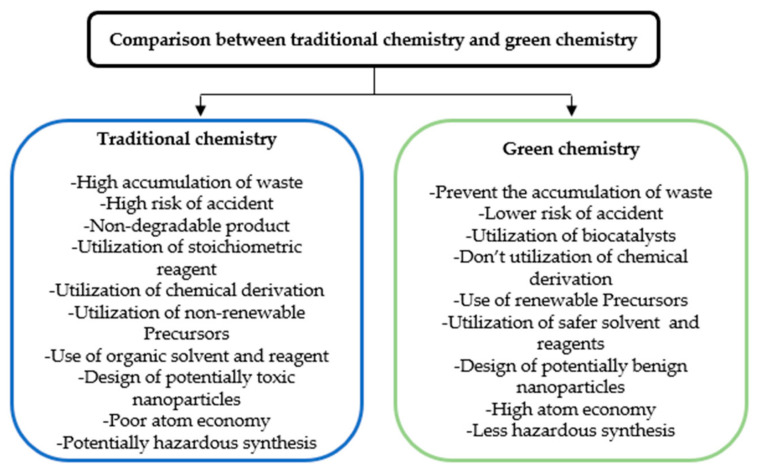
Comparison of green synthesis and traditional synthesis methods. Elaboration from Ref. [10].

Classical chemical and physical approaches involve high energy consumption and the use of toxic solvents and chemicals during the process, which results in environmental concern [7]. In contrast, the green synthesis of nanoparticles requires non-toxic, environment-friendly, and safe reagents [4] to produce simple, cheap, and more stable nanoparticles [8]. To this aim, plants or plant extracts and various microorganisms such as algae, bacteria, yeast, and fungi are used. Finally, to synthesize nanoparticles by biological method, proteins, enzymes, sugars, flavonoids, phenols, etc., function as the reducing and stabilizing agents [9]. A comparison between traditional chemistry and green chemistry is depicted in Figure 1.

Gas sensors have gained considerable importance for environmental and industrial atmosphere monitoring in the last years [11]. Gas sensing techniques are based on resistance measurement, gas and liquid chromatography, electrochemical and optical methods, as well as acoustic waves. Nevertheless, some sensors have several disadvantages: they are energy and time-consuming, large in size, expensive, and present slow response and low selectivity [12,13]. Therefore, special attention has been paid to chemoresistive sensors, which consist of metal oxides, conductive polymers, and carbon-based materials [14]. Among these materials, semiconducting metal oxides have been extensively explored and studied because they can have various valences, morphologies, and physical-chemical features [15]. They are more complex than pure metals, with bonding changing from ionic to highly covalent and metallic. Therefore, metal oxide nanoparticles are gaining considerable attention from industry to be used in various applications, including catalytic processes, electronics, sensors, magnetic storage media, and solar energy conversion [16,17]. Metal oxide semiconductors such as ZnO, CuO, Fe_3_O_4_, In_2_O_3_, and SnO_2_ have been extensively applied for the detection of volatile organic compound (VOC) gases and environmentally hazardous gases like NO_2_, NO, N_2_O, H_2_S, CO, NH_3_, CH_4_, SO_2_, and CO_2_ owing to their high sensitivity, low cost, thermal stability, easy production, etc. [18,19].

This review paper encompasses a detailed study of green synthesis methods and their merits and demerits. The application of green synthesized metal and metal oxide nanoparticles is mentioned hereunder. This paper also provides possible biological sources for the synthesis of the nanoparticles. In addition, the gas sensing mechanism of n- and p-type metal oxide semiconductors (MOS) sensors are comprehensively explained. Finally, this work also gives an insight into the effect of morphology and structure of MOS on their sensing performance. Furthermore, current literature will focus on comprehensive findings during recent developments in the biosynthesis of semiconductor metal oxides, namely, ZnO, CuO, Fe_3_O_4_, In_2_O_3_, WO_3_, Ce_2_O_3_ via plant extract, and bacteria, fungi, and algae to use in gas sensing applications.

## 2. Green Synthesis of Nanoparticles (NPs)

**Figure 2:**
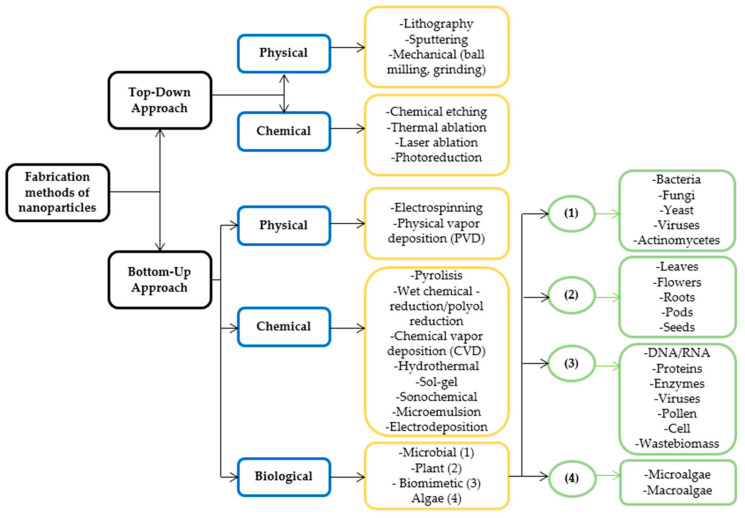
Top-Down and Bottom-Up approaches for synthesizing nanoparticles, (1) Microbial; (2) Plant; (3) Biomimetic; (4) Algae. Elaboration from Ref. [24].

Findings have demonstrated that special attention has been given to replace traditional physical and chemical methods for synthesizing metal and metal oxide nanoparticles with biosynthesis processes termed green synthesis or biological methods [20,21]. In general, there are two principal methods for the synthesis of nanoparticles named the “top-down” and “bottom-up” methods [22], as presented in Figure 2.

In the top-down approach, nanoparticles are produced by reducing the size and decomposing the bulk materials into fine particles [23] through various physical and chemical routes such as lithography [24], sputtering [25], thermal evaporation [26], pulsed laser ablation [27], photoreduction techniques [28], chemical etching [29] and mechanical (milling, and grinding) [30]. Nevertheless, the imperfection of the produced surface structure is mentioned as the main disadvantage of this technique [31]. In the bottom-up method or self-assembly method, the nanoparticles are synthesized by joining smaller units such as atoms and molecules [32,33] via chemical vapor deposition (CVD) [34], sol-gel [35], co-precipitation [36], hydrothermal synthesis [37], electrodeposition [38], radiation-induced [32], pyrolysis [39] and wet chemical routes [40,41]. However, there are some challenges when using these methods, including high energy consumption, long reaction time, use of hazardous and toxic substances, and non-eco-friendly by-products.

As shown in Figure 2, the green synthesis method is categorized as a bottom-up approach. Different mechanisms are involved in this process, including micro-organic content based mechanism, plant extract mediated mechanism, microwave-based synthesis, vitamin-based green synthesis, photo-catalysis, hydrothermal mechanism, ultrasound-based synthesis, magnetic synthesis, various solvents/catalysts in greener routes, supercritical fluids (SC), and ionic liquids (ILs) [42].

In contrast to the chemical reduction process, in the biosynthetic route, plant extracts are a replacement for expensive and toxic reagents. Some merits and drawbacks of the green synthesis method are summarized in Table 1.

Moreover, green synthesized metal and metal oxides nanoparticles have various applications in antimicrobial, biomedical, targeted drug delivery, and sensors, as presented in Table 2.

### 2.1. Biological Sources for Green Synthesis of Nanoparticles

Traditional physical and chemical methods for synthesizing metallic nanoparticles need high energy, highly toxic reducing, and stabilizing agents leading to detrimental effects on both humans and sea life. In contrast, the green synthesis of these particles includes a one-step, cost-effective, and eco-friendly approach that can initiate the reaction with low energy. The biological routes to synthesize metal and metal oxide nanoparticles have been focused on reducing agents such as bacteria, fungi, yeast, algae, and plant extracts considered in biocompatible and large-scale production [5,45].

#### 2.1.1. Bacteria-Mediated NPs Generation

Bacterial species have been extensively studied in commercial biotechnological applications, including bioremediation, bioleaching, and genetic engineering due to their relatively facile manipulation [46,47]. It is found that bacteria are a good candidate for preparing metallic and novel nanoparticles [42]. In particular, Prokaryotes and Actinomycetes have been widely utilized to synthesize metal and metal oxide nanoparticles. Certain bacteria with specific shapes and sizes, such as *Bacillus cereus*, *Lactobacillus casei*, *Aeromonas* species, *Pseudomonas proteolytica*, *Escherichia coli*, etc., can be used to synthesize silver-based nanomaterials by biological reduction [46]. Moreover, some other bacteria agents such as *Shewanella* alga, *Bacillus subtilis* 168, *Plectonema boryanum* UTEX 485, and *Rhodopseudomonas capsulata* were applied for the synthesis of gold nanoparticles [48].

#### 2.1.2. Fungi-Mediated NPs Generation

Biosynthesis of metal and metal oxide nanoparticles using fungal species is considered an efficient method to generate monodispersed NPs with suitable morphologies. In comparison with other organisms, fungi are better biological agents for synthesizing NPs (such as silver and gold) due to their intracellular enzymes, proteins, and reducing components on the surface of their cell. Mainly, they have the ability to produce a larger number of NPs with respect to bacteria. The formation mechanism of metal nanoparticles can be explained by an enzymatic reduction in fungal cells or their cell wall [49,50,51]. The use of fungal extracts for synthesizing nanoparticles has several benefits over bacterial extracts.

**Figure 3:**
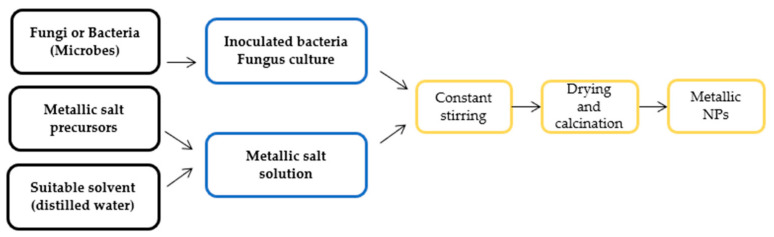
Microbe-mediated synthesis of nanoparticles. Elaboration from Ref. [53].

Fungal extracts for the synthesis of NPs have a significant advantage over bacterial extracts. They are economically viable, have large-scale production and easy extraction, are eco-friendly, and possess a large surface area. Additionally, it has been reported as a good source of metabolites and enzymes with the ability to reduce bulk salts into elemental ions, which are essential for nanoparticle synthesis [52]. The green synthesis of nanoparticles via a microbe-mediated route is displayed in Figure 3.

#### 2.1.3. Yeast-Mediated NPs Generation

Yeasts are known as unicellular microorganisms in eukaryotic cells, and only 1500 species have been identified. Numerous studies have been conducted on the synthesis of nanoparticles and nanostructures using a variety of yeast species through yeast-reducing enzymes intracellularly or extracellularly. For example, gold and silver nanoparticles were synthesized using a silver-tolerant yeast strain and *Saccharomyces cerevisiae* broth [54,55,56].

#### 2.1.4. Algae-Mediated NPs Generation

Algae is rarely used as a biofactory for synthesizing metallic NPs [57,58]. Singaravelu et al. [59], for the first time, produced extracellular gold nanoparticles with high stability by applying a marine alga (*Sargassum wightii*). Furthermore, they reported the green synthesis of palladium and platinum nanoparticles using their metallic chloride-containing salts. Fawcett et al. [60] investigated the green synthesis of three metal oxide nanoparticles, copper oxide, ferric oxide, and zinc oxide, via marine algae.

#### 2.1.5. Plants-Mediated NPs Generation

Among biological components, plants have attracted tremendous attention to the synthesis of nanoparticles. Their excellent properties include inexpensive cost, stability, simplicity, safety handling, low energy consumption, low consumption of toxic chemicals, rapid reaction time, and a wide range of biomolecules. Typically, plants have excellent potential to reduce metallic salts into nanoparticles due to some biomolecules such as carbohydrates, proteins, and coenzymes. Various parts of plants also have the ability to accumulate heavy metals inside their parts. Moreover, the plant extracts can control the synthesis of nanoparticles to obtain well-defined morphologies and size using only one step with a high yield synthesis [22]. Therefore, extensive attention has been paid to the biosynthesis of nanoparticles using various parts of plants such as leaves, roots, flowers, and fruits as a facile, efficient, cost-effective, and alternative route to traditional production methods [53]. Gold and silver were the first biosynthesized nanoparticles using plant extract [58,61,62,63].

## 3. Factors Affecting on Green Synthesis of Nanoparticles (NPs)

Different parameters affect the characteristics of biosynthesized nanoparticles and control their stability [64], including pH value [65], calcination and reaction temperature [66], concentration [67], pressure, solvent type, and contact time [68]. The pH of the reaction medium has a key role in nanoparticle formation. Different concentrations of hydrogen ions cause diversity in the shape and size of nanoparticles. It was found that larger particles can be produced at lower acidic pH values. Rod-shape Au nanoparticles were produced using *Avena sativa* at pH 2 and 3 with particles size of 25–85 nm and 5–20 nm, respectively. In comparison, spherical silver nanoparticles were synthesized by *Cinnamomum zeylanicum* bark extract at a higher pH value.

Temperature plays a stimulating effect in the synthesis of metallic nanoparticles owing to its impact on the shape and size of NPs. Synthesis of gold nanoparticles using *Cymbopogon flexuosus* leaf extract at lower reaction temperature led to the formation of nanotriangles particles, while spherical nanoparticles and triangle nanoparticles formed at higher reaction temperature [64].

Pressure has a remarkable effect on the synthesis of metallic nanoparticles due to its impact on their size and shapes. Some studies [69] have reported that, at ambient pressure conditions, the metal ions reaction occurs at a faster rate.

The time duration of incubation for the reaction of nanoparticles significantly affects their quality, morphology, and characteristics. Incubation of nanoparticles for a long time can cause aggregation or shrinkage, leading to a decrement in the potential of nanoparticles [64].

Nevertheless, the main factor is the presence of phytochemicals such as ascorbic acids, phenols, carboxylic acids, terpenoids, amides, flavones, aldehydes, ketones, etc. [70,71], which naturally exists in plant extracts. These mentioned components reduce metal salts to metal nanoparticles [45].

## 4. Gas Sensing Mechanisms

Chemoresistive sensors are the most extensively utilized gas sensors based on metal-oxides nanostructure [72]. These materials are semiconductors, and nonstoichiometry determines their main charge carriers, as perfectly stoichiometric compounds are too resistive [73]. The n-type semiconductivity is due to the formation of anions (oxygen) vacancies or to the substitution with higher valence cations which increase the number of free electrons [74,75]. On the contrary, the p-type semiconductivity can be attributed to holes generated either by the addition of interstitial anions or the substitution with lower valence cations [75]. Based on the electronic structure, metal oxide semiconductors can be categorized into two types as follows [76]:

- transition-metal oxides (like hematite, cobalt tetraoxide, nickel oxide, titanium oxide, tungsten oxide, etc.),
- non-transition-metal oxides, including pre-transition-metal oxides (such as alumina, magnesium oxide, etc.) and post-transition-metal oxides (zirconia, tin (IV) oxide, etc.).

The electronic structure controls the gas sensitivity of metal oxides. The pre-transition-metal oxides are seldom used for gas sensors due to their wide band gap and disability in the formation of both electrons and holes. In contrast, the gas sensing application of the transition-metal oxides and post-transition-metal oxides is guaranteed by electronic configurations [76]. Typically, the gas molecules adsorption on the sensing material’s surface is the operating principle of gas sensors based on metal oxide semiconductors. At 100–500 °C, when the sensor is exposed to air, oxygen molecules adsorb onto the surface of the material. The chemisorption of oxygen from the gas phase creates extrinsic surface acceptor states (O_2_^−^, O^2−^, and O^−^), immobilizing the conduction band electrons from the regions near the surface of the semiconductor [77]. The oxygen species on the surface of metal oxide can be physically or chemically adsorbed in function of the temperature. Below 150 °C, the molecular form dominates, and O_2_^−^ is physically absorbed (Equation (1)), whereas, above this temperature, the ionic species O^−^ (Equation (2)) and O^2−^ (Equation (3)) prevail, at temperatures below 250 °C and above 450 °C, respectively [77].

(1) $$O_{2\;\left(\mathrm{ads}\right)}+é\to O_{2\;\left(\mathrm{ads}\right)}^{-}$$

(2) $$O_{2\;\left(\mathrm{ads}\right)}^{-}+é\;\to 2O^{-}$$

(3) $$O_{2\;\left(\mathrm{ads}\right)}^{-}+é\;\to {\;O}_{\left(\mathrm{ads}\right)}^{2-}$$

**Figure 4:**
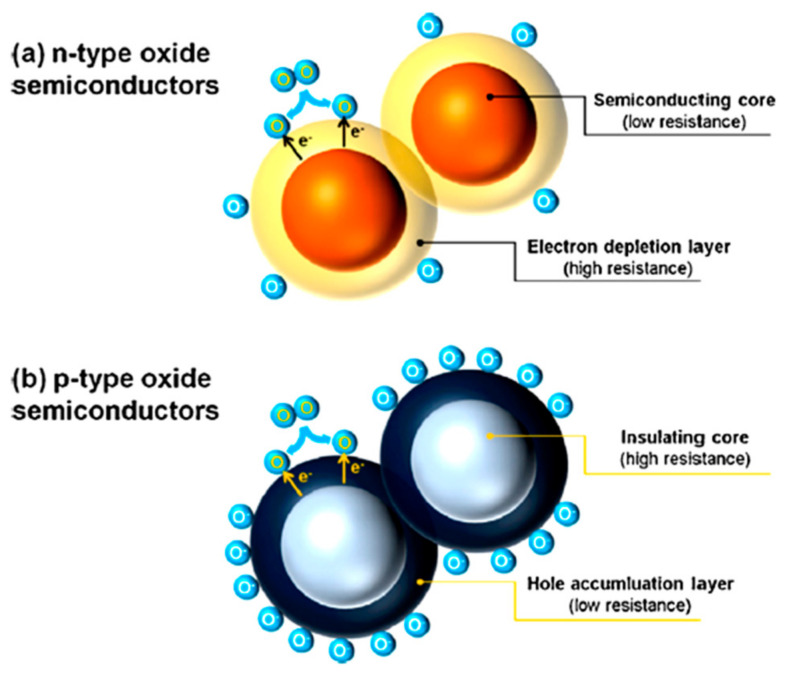
The electronic core-shell structures formation in metal oxide semiconductors: (**a**) n-type; (**b**) p-type. Reprinted from Ref. [78] with permission of Sensors & Actuators: B. Chemical, 2014.

Due to the adsorbed oxygen molecules from the air (under the ambient condition), a resistive electron depletion layer (EDL) forms on the surface of the sensing materials (Figure 4a). In the p-type MOS, the adsorption of oxygen anions causes the formation of a hole accumulation layer (HAL) at the outer surface (Figure 4b) [78].

The gas sensing behavior of n-type and p-type MOS are different in function of the target gases, as listed in Table 3 [79].

**Figure 5:**
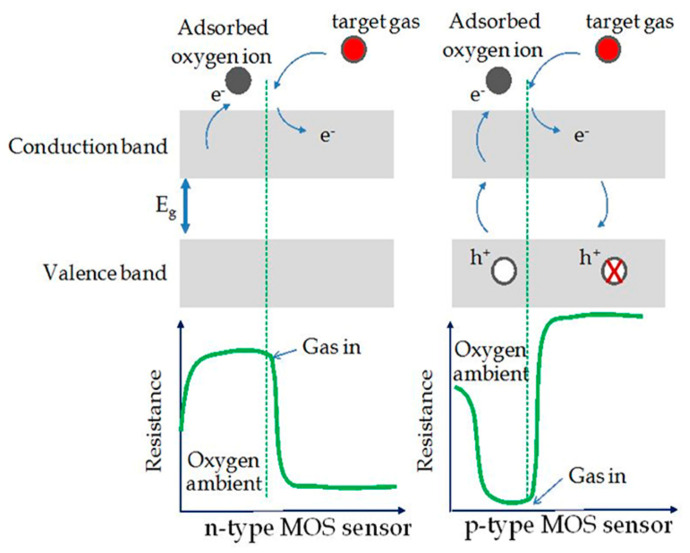
A schematic for the change of sensor resistance when exposed to the reducing gas in the cases of n–type and p–type metal oxide semiconductor sensors. Reprinted from Ref. [81].

In the case of n-type MOS semiconductors, the outer resistive shell is responsible for the sensor resistance when this sensor is exposed to air. Nevertheless, if samples are exposed to a reducing gas (Figure 5), the reaction between the ionic oxygen species and the gas will occur. Subsequently, the captured electrons will be released in the conduction band, leading to a decrement of the material resistance. On the contrary, if any oxidizing gas is introduced, the material will be further oxidized, and the thickness of the EDL, as well as the resistance of the material, will further increase. In addition, if the grain size of the sensing material is less than twice the Debye length (L_D_, Equation (4)), the EDL will extend to the entire material, and then the electrical change (and thus, the sensor response) will be maximum.

(4) $$L_{D}=\sqrt{\frac{{\varepsilon K}_{B}T}{q^{2}N_{D}}}$$

where: ε is the dielectric constant, k_B_ is the Boltzmann’s constant, T is the absolute temperature in Kelvin, q is the elementary charge, and N_D_ is the net density of dopants (either donors or acceptors).

P-type semiconductors present an opposite behavior with respect to n-type ones. In this case, both the resistive core and the p-type shell determine the conductivity of the sensing materials [80]. Nevertheless, if the particles of MOS are larger than twice the thickness of the HAL, any slight change in the concentration of holes in the shell layer due to electron-hole recombination will not change significantly the resistance value. Therefore, the conduction of p-type semiconductors mainly occurs within semiconducting shells.

A metal-oxide semiconductor gas sensor contains a sensing layer, two or more electrodes, and a heater to react with the target gas, detect the resistance changes, and provide operating temperature, respectively [82,83]. The sensor response is defined in Table 3 based on the type of metal oxides and target gases. The response and recovery time are expressed as the time the sensor takes to obtain 90% of the total resistance change in the case of target gas adsorption and desorption, respectively [79]. Some other parameters such as selectivity, operating temperature, and stability are remarkable parameters of gas sensors. At a specific working temperature, the MOS sensors present various responses toward different target gases [84]. Thus, with resistive semiconductor gas sensors, the chemical selectivity can be obtained by operating the sensor at different temperatures [84]. A sensor with good selectivity means that it is able to detect the target gas even in a mixture of gases. According to the reported studies, MOS-based gas sensors are mainly able to detect a gas owing to the change in the electrical signal caused by the gases [85].

## 5. Metal Oxide Nanoparticles (MO NPs)

**Figure 6:**
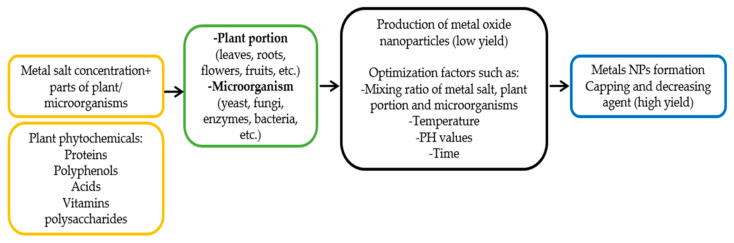
Green synthesis of metal oxide using various plants portions and microorganisms. Elaboration from Ref. [86].

Extensive efforts have been provided to convert metal salts into metal oxides using plant extracts (Figure 6) [86]. One of the significant aspects of the use of plants for integrating nanoparticles is the biological variety of plant extracts, as well as the easy access to numerous phytochemicals in different plant portions. Additionally, the produced nanoparticles present enhanced features and applications with suitable manipulation. Therefore, most demand has been focused on green nanotechnology [22,87].

Table 4 shows the biological synthesis of metal oxide nanoparticles using different biological agents.

With the development of technology and increasing demand for precise information in different fields, sensors have drawn interest in various applications, including aerospace, medical diagnosis, ocean exploring, industrial manufacturing, bioengineering, and environmental protection [129]. A wide range of metal oxide materials, including zinc oxide, titanium dioxide, tin (IV) oxide, indium (III) oxide, copper (II) oxide, nickel (II) oxide, and iron (II, III) oxide, have been considered as promising materials for detecting hazardous and polluting gases such as H_2_, NH_3_, NO_2_ as well as biosensing of glucose, hydrogen peroxide, etc. In addition, it has been proven that metal oxide composites consisting of two or more metal oxides can increase gas sensor sensitivity [130]. Table 5 illustrates the various metal oxide composites and mixed metal oxides used as a sensor for detecting different gases.

### Effect of Morphology and Nanostructure of MOS on Gas Performance

The morphology of metal oxide semiconductors leads to their unique characteristics that are critical for their further applications, such as gas sensing. The synthesis approach mainly controls the morphology and structures of materials. Therefore, the selection of the preparation method is determinant to obtain the sensing materials with desirable properties such as morphology, grain and particle size, the crystal and electronic structures, the network connection, and physical characteristics (bandgap) [132].

As well-known and previously discussed, in metal oxide semiconductors, the electronic transfer occurs with the surface adsorption and desorption of gas molecules, leading to a change in conductivity. Hence, the shape and size of nanostructures have a significant role in these changes. Sensing materials with a larger surface area possess more active sites for adsorption and reaction of gas molecules. If the material’s structure includes some conductive channels, these facilitate electron transmission and improve electron mobility, leading to faster response/recovery speed. In addition, nanosheet structures have demonstrated high potential in gas sensing applications due to the large specific surface area and extremely thin nanosheets. Another significant factor in the adsorption of the target gas is the porous morphology [130]. Indeed, the porous channel structure presents a transmission path for the target gas and provides large pores. Therefore, porous channels improve the diffusion of gas molecules in metal oxide gas sensors by providing a large surface area and consequently increasing the number of active sites for gas molecules adsorption.

Despite the unique merits of one (1D) and two dimensional (2D) structures, they present higher stresses and inevitably agglomerate that are harmful to the stability of the sensor. In comparison, 3D structures such as 3D flower-like morphology overcome these disadvantages and inherit the benefits of 1D and 2D structures like high specific surface area. Besides these, the solid structure consists of one adsorption layer. In contrast, the hollow structure has two adsorption layers, both inside and outside, so that sensors can achieve more active sites for target gas adsorption with better gas sensitivity [130].

**Figure 7:**
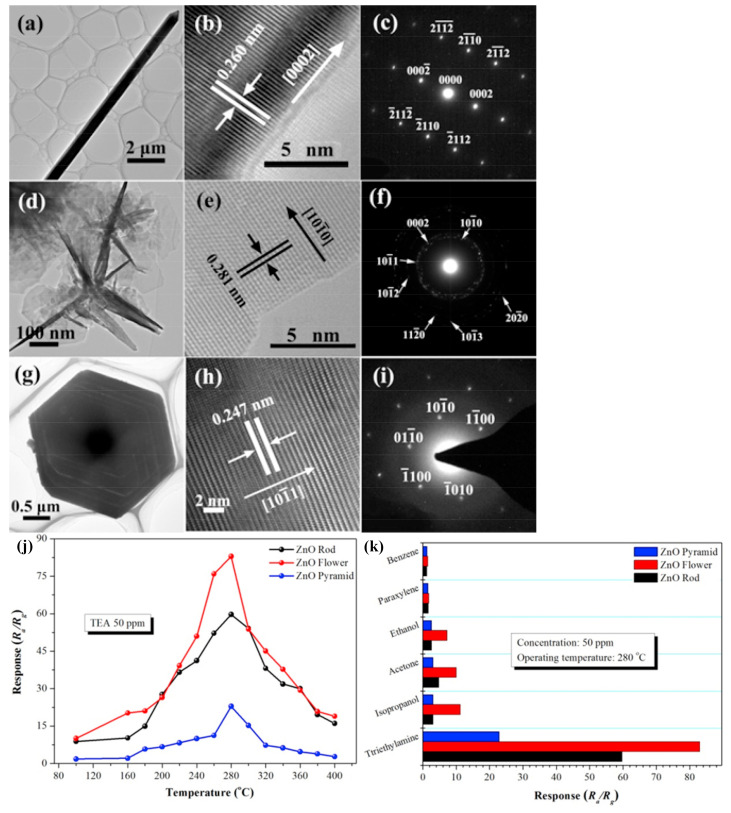
(**a**–**c**) TEM image, corresponding HRTEM image and SAED pattern of ZnO rod; (**d**–**f**) TEM image, corresponding HRTEM image and SAED pattern of ZnO flower; (**g**–**i**) TEM image, corresponding HRTEM image and SAED pattern of ZnO pyramid; (**j**) Gas sensitivity of ZnO versus working temperature towards 50 ppm of triethylamine; (k) Selectivity comparison of ZnO sensors under 50 ppm of various target gases at 280 °C, Reprinted from Ref. [138] with permission of Elsevier Ltd., 2017.

Researchers have studied the influence of various morphologies on the gas sensing performance of ZnO [133,134,135]. For example, Godse et al. [136] prepared ZnO nanorods and investigated the sensing performance under NO_2_ gas. The ZnO nanorods exhibited great gas sensitivity with a sensor’s response of 70% and response and recovery times of 16 s and 200 s, respectively, toward 5 ppm of NO_2_. Van Duy et al. [137] synthesized zinc oxide nanosheets with a thickness of 15 nm and a sensor’s response of 76% under 0.5 ppm NO_2_. Nevertheless, other scholars prepared porous ZnO nanosheets with a porosity of about 16% and an average pore size of 60 nm that showed a response of 2.93% under 0.5 ppm NO_2_ [133]. Li et al. [138] synthesized three kinds of typical ZnO microstructures: rods, flowers, and pyramids. They reported that the dominant facets for ZnO rods, flowers, and pyramids were (100) plane, (0001) plane, and (101) plane, respectively (Figure 7a–i). The ZnO with the morphology of micro flowers exhibited the highest response (83%), the best selectivity to triethylamine (TEA) as well as short response/recovery times (8 s/23 s, respectively) at the operating temperature of 280 °C (Figure 7j,k).

**Figure 8:**
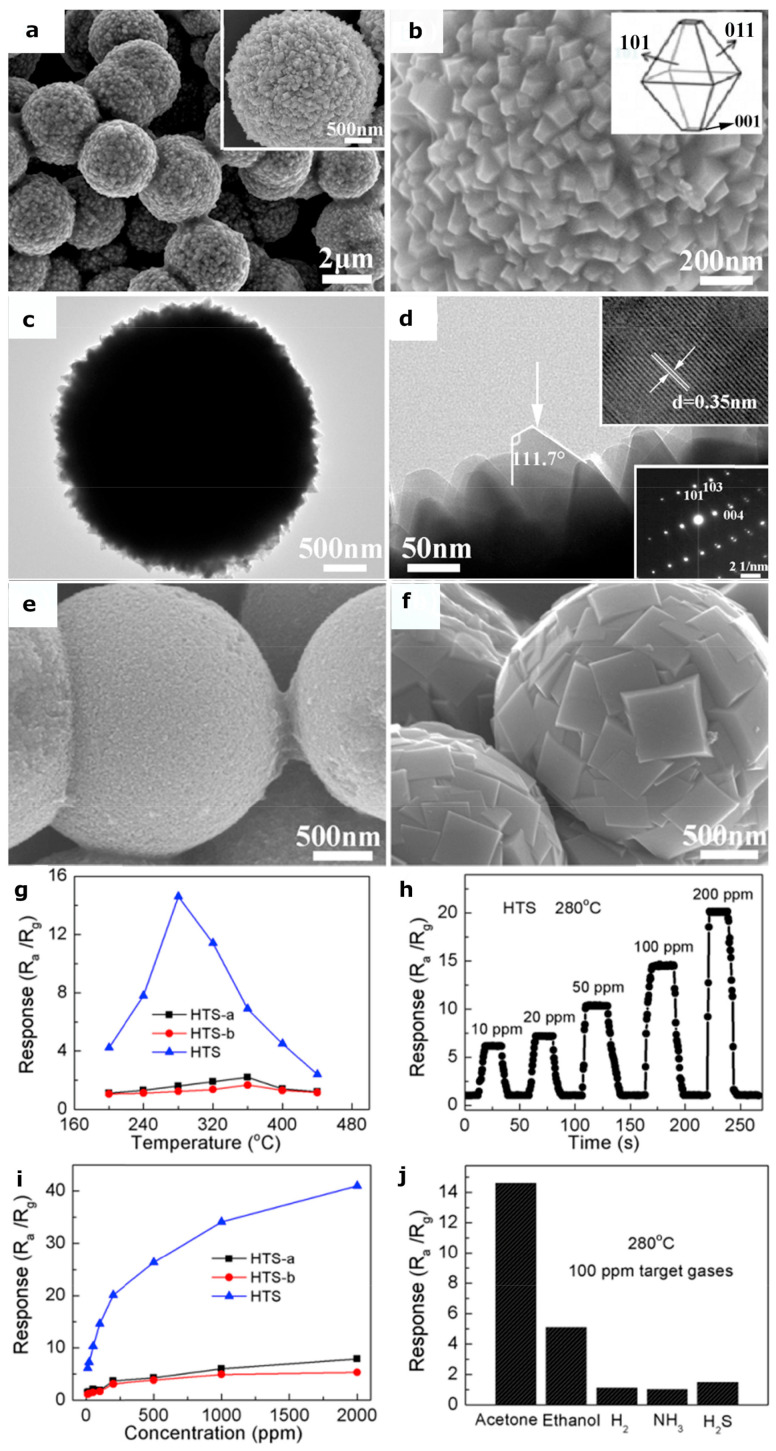
(**a**,**b**) FESEM images and (**c**,**d**) TEM image of HTS; (**e**) FESEM images of HTS-a; (**f**) HTS-b; (**g**) Response of three TiO_2_ sensors versus various working temperatures to 100 ppm acetone; (**h**) Gas response of HTS under different concentrations of acetone at 280 °C; (**i**) Response of the three TiO_2_ sensing materials to 10–2000 ppm of acetone; (**j**) Selectivity of HTS for the detection of 100 ppm of various target gases, Reprinted from Ref. [139] with the permission of Elsevier Ltd., 2017.

**Figure 9:**
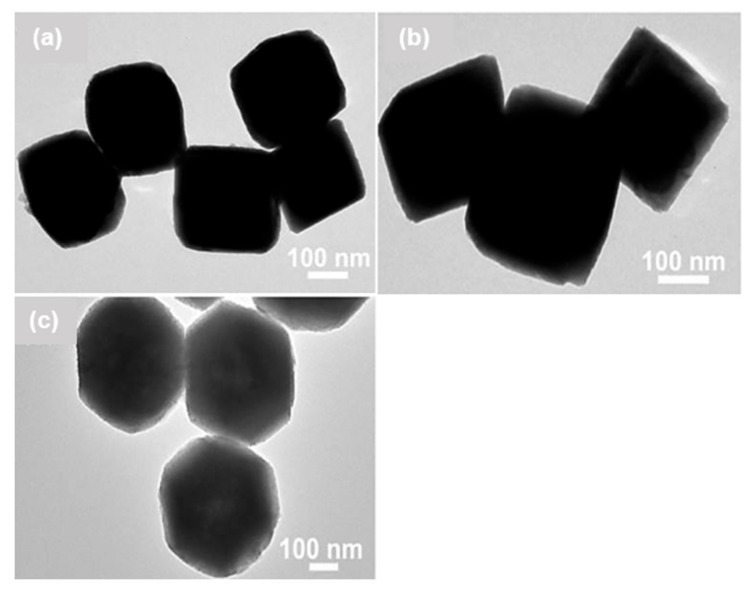
TEM images of: (**a**) Cu_2_O-cube; (**b**) Cu_2_O-octahedron; (**c**) Cu_2_O truncated octahedron, Reprinted from Ref. [140], with the permission of Elsevier Ltd., 2017.

Facet-controlled synthesis is one of the most promising strategies to develop high-performance sensors, because some facets with higher surface energy, dangling bonds and densities of atoms respect to others usually show a much higher chemical activity. To this aim, Yang et al. [139] synthesized anatase titanium dioxide hierarchical microspheres (HTS, Figure 8a–d) using a hydrothermal method. They studied the effect of various morphologies such as nano TiO_2_ microspheres (HTS-a, Figure 8e) and mirror-like plane-covered TiO_2_ microspheres (HTS-b, Figure 8f) on the sensing performance. The FESEM and HRTEM images showed (Figure 8a–d) that the microspheres of HTS were exposed with (001) and (101) facets on the surface. Concerning HTS-a and HTS-b, the HTS sample exhibited excellent sensing performance of 14.6 to 100 ppm to acetone vapor with a lower working temperature of 280 °C along with a rapid response/recovery speed and an excellent selectivity (Figure 8g–j). In addition, the response of HTS-based sensor to 100 ppm of acetone was much higher respect to when under 100 ppm ethanol, H_2_, NH_3_ and H_2_S (Figure 8j). Wang et al. [140] prepared three different kinds of copper oxide powders: cube, truncated octahedron, and octahedron, using a hydrothermal method and investigated their sensing performance. Ascorbic acid was used as the reducing agent and polyvinylpyrrolidone (PVP) was added in different amounts to control the microstructure of both synthesized octahedron powders. The obtained samples had a highly regular polyhedral structure with approximately 250 nm, 450 nm, and 400–600 nm in sizes for the cube, octahedron, and truncated octahedron of Cu_2_O, respectively (Figure 9a,b,c).

**Figure 10:**
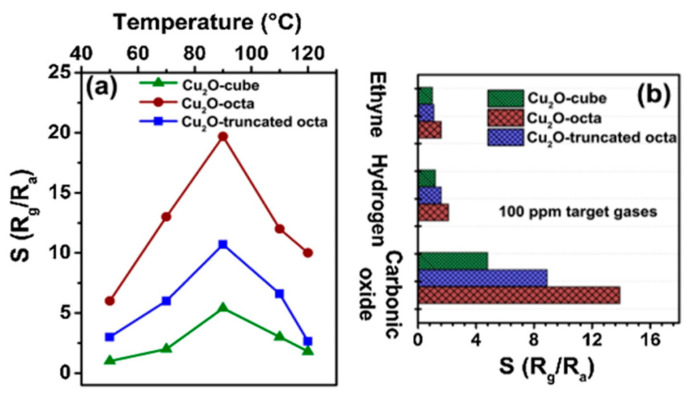

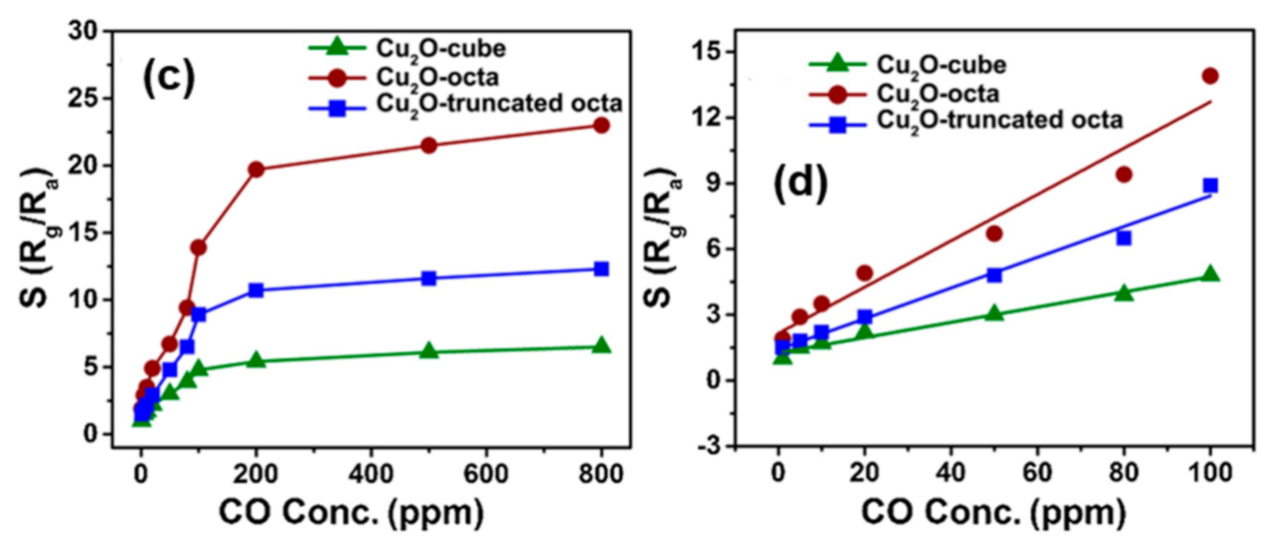
(**a**) The relationship of working temperature and CO-sensing response of three Cu_2_O particle-based sensors to 200 ppm CO; (**b**) Sensor responses of Cu_2_O sensors to 100 ppm of CO, H_2_, and C_2_H_2_ at 90 °C; (**c**) Response versus CO concentration of Cu_2_O sensors at 90 °C; (**d**) The corresponding sensor response curves to CO concentration from 1 to 100 ppm; The straight line show fits the data. Reprinted from Ref. [140] with the permission of Elsevier Ltd., 2017.

The sensing response of the three obtained Cu_2_O sensors were evaluated in the presence of 200 ppm CO at different working temperatures (Figure 10a). The maximum CO response value was obtained by Cu_2_O-octahedron (19.7) at 90 C which was 3.6 and 1.8-times higher than that of the Cu_2_O-cube (5.4) and Cu_2_O-truncated octahedron (10.7). Besides these, the Cu_2_O-octahedron particles-based gas sensor exhibited a response of 13.9 to 100 ppm of CO, whereas the sensing responses towards C_2_H_2_ and H_2_ were negligible (Figure 10b). The response to different concentrations of CO (1 to 800 ppm) showed that all sensor responses enhanced with increasing the CO concentration (Figure 10c). Furthermore, the authors reported a response of 1.9 for the Cu_2_O-octahedron particles-based sensor toward 1 ppm CO at 90 °C (Figure 10d). According to the obtained results and excellent linear dependence of response on the CO concentration, they suggested that the sensors based on Cu_2_O-octahedron particles are promising candidates to low concentration CO detection. Last, but not least, the relationship between the surface catalytic properties of the exposed crystal planes and surface composition/structure of various Cu_2_O particles is of fundamental importance to understand the sensing reactions from the point of view of materials’ chemical activity.

**Figure 11:**
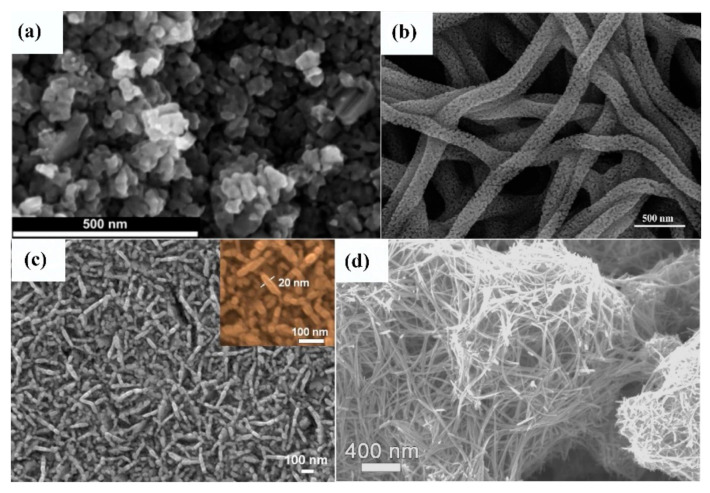
SEM images of: (**a**) WO_3_ nanoparticles; (**b**) WO_3_ nanofibers; (**c**) WO_3_ hollow nanosheet array; (**d**) WO_3_ nanomesh, reprinted from Refs. [141,142,143,145], with permission of Elsevier Ltd., (**b**,**c**).

Matovic et al. [141] prepared the nanoparticle WO_3_ (Figure 11a) with two crystal types (monoclinic phase γ-WO_3_ and hexagonal phase h-WO_3_). These Authors reported a sensor response of 450 to 1 ppm NO_2_ at room temperature. The enhanced sensor response was attributed to the electron and hole transfer from h-WO_3_ to the γ-WO_3_ phase. Zhang et al. [142] reported a sensitivity of 101.3% and response/recovery times of 125/231 s, respectively, for WO_3_ nanofibers (Figure 11b) toward 3 ppm NO_2_. In another study, WO_3_ porous nanosheet arrays (Figure 11c) with a thickness of 20 nm exhibited a high response of 460% under 10 ppm NO_2_ at 100 °C [143]. The WO_3_ hollow sphere sensor obtained a significant sensing performance towards sub-ppm NO_2_ [144]. The experimental results showed a sensor response of 1.5~26% in the range 50 ppb~1 ppm of NO_2_ at room temperature, which was attributed to the large specific surface area, high concentration of oxygen vacancy, and hollow structure. Liu et al. [145] synthesized a nanomesh assembled from WO_3_ nanowires (Figure 11d) to detect low concentrations of NO_2_ at lower temperatures. They reported a sensor response of 1.25% to 50 ppb NO_2_ at 160 °C.

## 6. Green Synthesis of Metal Oxide Semiconductors

### 6.1. Zinc Oxide Nanoparticles (ZnO NPs)

The investigation of zinc oxide nanoparticles has increased because of their vast applications in solar cells, photovoltaic, drug delivery systems, gas sensors, field emission devices, coating, electrochemical, antibacterial, capacitors, and cosmetics [22,146,147]. ZnO is an n-type semiconducting metal oxide (SMO) having piezoelectric and pyroelectric characteristics, long life, as well as high sensitivity to various target gases. The ZnO nanoparticles present different semiconducting features as they possess high exciton bonding energy and a wide bandgap with 60 meV and 3.37 eV, respectively. These characteristics have made ZnO important to extensively apply as a gas sensing material in houses and industrial environments to detect toxic, dangerous, explosive, and greenhouse gases [148] and for air quality assessment. The ZnO NPs can have various morphologies such as nanoflower, nanorod, nanoflake, nanobelt, nanosheets, and nanowire.

**Figure 12:**
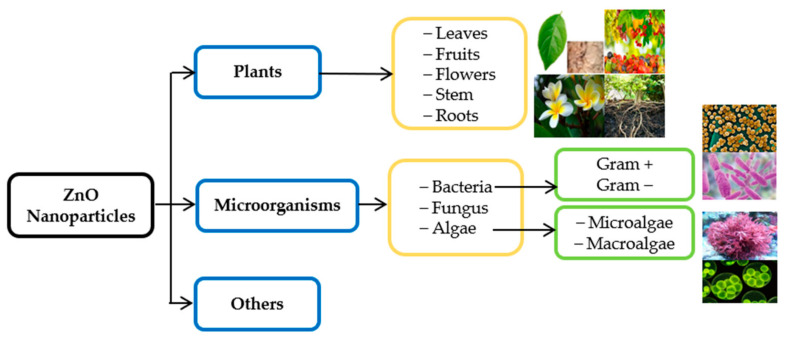
Synthesis of ZnO NPs by using various biological species. Elaboration from Ref. [156].

A wide range of studies has been conducted on green synthesized ZnO NPs in biomedical applications, gas sensing, purification of water, and optical devices [149,150]. Numerous investigations have dealt with the green synthesis of ZnO NPs using various plants, bacteria, fungus, algae, etc. (Figure 12). Several studies have reported the biological synthesis of ZnO via fungi (*Fusarium keratoplasticum* A1-3, *Aspergillus terreus*) [151,152] and bacteria (*Halomonas elongata* IBRC-M 10,214, *Sphingobacterium thalpophilum*) [153,154]. Furthermore, Sanaeimehr et al. [155] obtained ZnO nanoparticles with a particle size of 30–57 nm by *Sargassum muticum* algae extraction.

**Figure 13:**
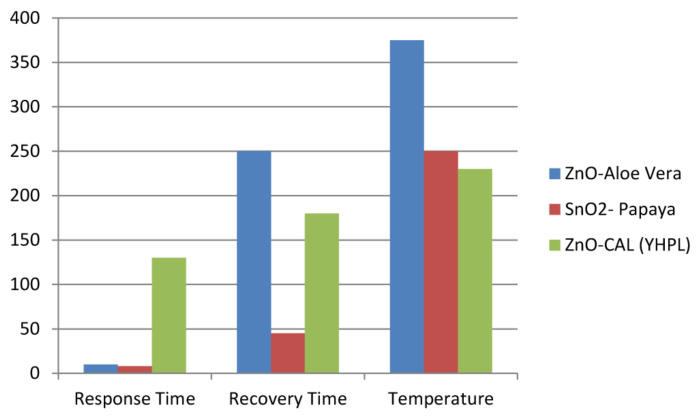
Comparison of response/recovery time (in second) and temperature (in °C) for ZnO-Aloe vera, SnO_2_-Papaya, and ZnO-CAL (YHPL). Reprinted from Ref. [86] with permission of Emerald Publishing Ltd., 2021.

Sabir et al. [157] reported the synthesis of ZnO NPs using milky latex of *Calotropis procera*, rice as soft biotemplate, and leaf extract of various plants such as *Coriandrum sativum*, *Calotropis gigantea*, *Acalypha indica*. Recently, Kumar et al. [158] successfully synthesized biodegradable and green chitosan/PVP (GHP) polymeric substrates using a facile and low-cost solvent casting method. Subsequently, ZnO film was deposited on the GHP substrate through an easy drop-casting technique. These Authors reported 24% and 46% sensor response against 0.5% and 2% H_2_ gas, respectively, at an operating temperature of 150 °C. Their experiments demonstrated the repeatability properties of GHP/ZnO towards 0.5% H_2_ gas at 150 °C with a repeatable sensor response over three cycles. Jadhav et al. [86] developed sensors for hybrid vehicles with the green synthesized ZnO and SnO_2_ powders. They prepared hexagonal and spherical/hexagonal ZnO nanoparticles from zinc nitrate hexahydrate using *Aloe vera* and *Young Harbara* Plant Leaves (YHPL), respectively. Figure 13 exhibits the gas sensing performance for ZnO-Aloe vera, SnO_2_-Papaya, and ZnO-CAL (YHPL).

Sensing properties of ZnO-Aloe vera showed the response time, recovery time, and sensitivity of 8–10 s, 230–250 s, and 48%, respectively, toward liquefied petroleum gases (LPG) in the operating range from 225 to 375 °C, while ZnO-CAL (YHPL) was used to detect NO_2_ gas with response time, recovery time, and sensitivity of 140–150 s, 150–200 s, and 38.2%, respectively, in the temperature range from 220 to 240 °C. Moreover, the LPG gas sensing properties showed the response time, recovery time, and sensitivity of 5–10 s, 25–50 s, and 56.25%, respectively, for SnO_2_-Papaya in the operating range from 200 to 250 °C.

Furthermore, scientists have studied ZnO nanoparticles in the biosensing field too. Alam et al. [157] synthesized NiO-CuO-ZnO nano metal oxides using a solution of *Centella asiatica* plant extract as a green fuel by combustion approach and examined their photocatalytic properties. The sensing ability of these metal oxides was also investigated as a glucose sensor. The ZnO nanoparticles were synthesized by *Carica papaya* seed extract and studied as an electrochemical biosensor for the detection of silymarin by Sharma et al. [159]. In another study, the sensing characteristics to ethanol of ZnO NPs were investigated by Joshi et al. [160] using *Ixora coccinea* leaf extract as a capping agent. The response time and recovery time were found equal to 24 s and 47 s, respectively, in the presence of 200 ppm of ethanol vapor.

**Figure 14:**
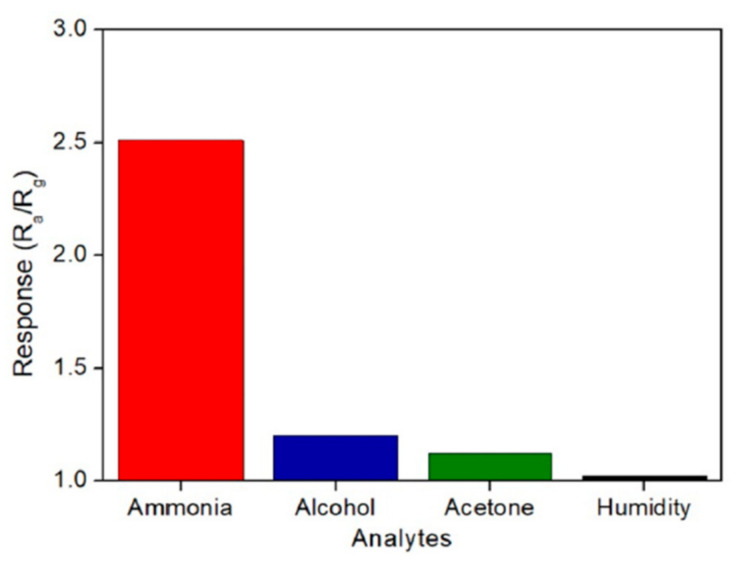
Selectivity of the synthesized sensor towards various test gases. Reprinted from Ref. [161] with permission of Elsevier Ltd., 2022.

Recently, in 2022, Nagar et al. [161] investigated the selectivity of the ZnO NPs sensors in the presence of alcohol, acetone, ammonia, and humidity. Hexagonal-shaped ZnO particles were obtained from zinc acetate dihydrate, soda, and *Parthenium hysterophorus* leaf extract. The presence of certain enzymes and proteins in the leaf extract acted as reducing, capping, and stabilizing agents for ZnO NPs. When dissolved in leaf extract, zinc acetate formed complex agents with proteins and enzymes, then, nucleation started and led to reverse micelle, which later reduced and formed NPs. Finally, ZnO NPs precipitated in the micelles’ core after NaOH addition. Enzymes and proteins in the leaves extract prevented clustering by shielding ZnO NPs [162,163,164]. From Raman and XPS investigations, the authors showed that the prepared powder was crystalline, oxygen deficient and possessed Zn interstitials, as well as a hexagonal wurtzite structure. They found also that the synthesized sensor was highly selective for the detection of ammonia at 80 °C (Figure 14).

**Figure 15:**
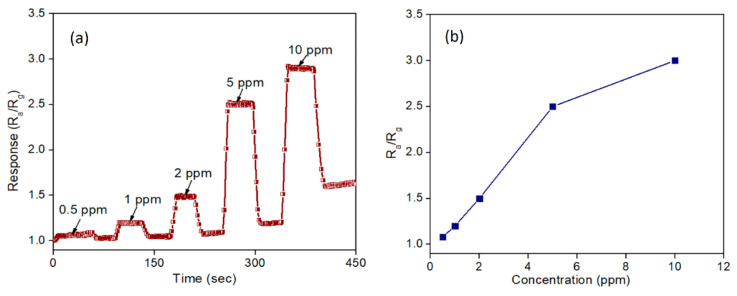
ZnO sensor response to ammonia gas in function of: (**a**) time and different gas concentration; (**b**) gas concentration. Reprinted from Ref. [161] with permission of Elsevier Ltd., 2022.

Furthermore, the sensor response was also assessed in various concentrations of NH_3,_ including 0.5, 1, 2, 5, and 10 ppm, as shown in Figure 15. At 0.5 ppm concentration, despite the sensor being active, it did not present a noticeable response. Moreover, the sensor characteristics were measured for 2 and 10 ppm of NH_3_ with response time and recovery time (7 s, 8 s) and (8 s, 14 s), respectively. Based on their experimental results, although the sensor response was approximately 300% at 10 ppm ammonia concentration, its recovery time was quite long (14 s). Hence, they reported excellent sensor characteristics at 5 ppm of NH_3_ with a response value of 2.5 and response/recovery time of 5 s and 8 s, respectively.

**Figure 16:**
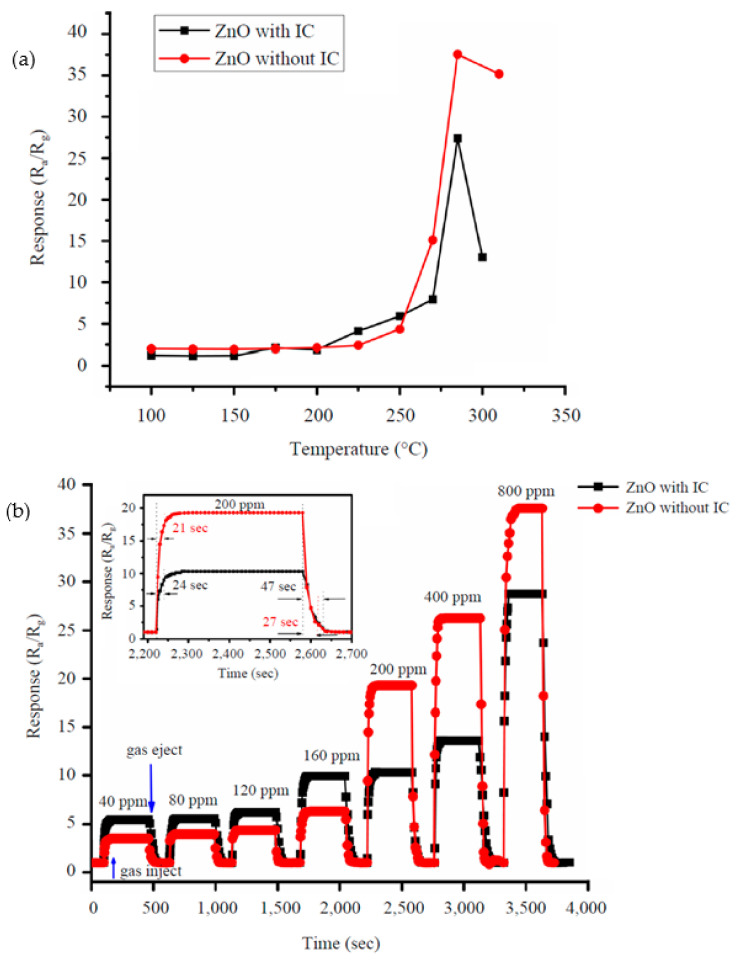
The gas-sensing performance of ZnO film: (**a**) at various temperatures; (**b**) various gas concentrations. Reprinted from Ref. [160].

Joshi et al. [160] studied the gas sensing characteristics of biosynthesized ZnO NPs using *Ixora coccinea* leaf extract (IC), zinc acetate dihydrate, and soda. A less aggregated ZnO powder was obtained due to the presence of IC extract acting as a capping agent. However, the crystallite size was comparable (23.08 nm for ZnO NP with IC and 22.02 nm for ZnO NP without IC). The authors compared the ethanol vapor sensing of ZnO samples with and without IC at various working temperatures towards 40–800 ppm of test gas. They found that the optimum temperature for both samples was 285 °C (Figure 16a). By increasing the ethanol concentration up to 800 ppm, there was a linear increment in the gas response (Figure 16b) up to an operating temperature of 285 °C. The sensor response was 28.7 and 37.4 for synthesized ZnO under 800 ppm of ethanol with and without IC, respectively. This difference in the gas response can be attributed to the change of morphology for synthesized samples from ZnO highly clustered to less aggregate clustered ZnO with and without IC, respectively. The response and recovery times reported were (24 s and 47 s) and (21 s and 27 s) for ZnO, with and without IC, respectively (the inset in Figure 16b).

**Figure 17:**
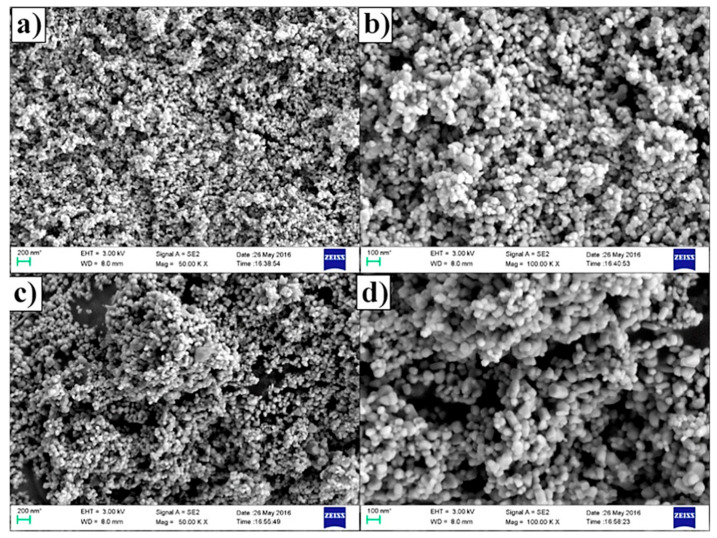
FE-SEM micrographs of zinc oxide nanoparticles: (**a**,**b**) biologically synthesis; (**c**,**d**) chemical synthesis. Reprinted from Ref. [166] with permission of Elsevier Ltd., 2017.

Zhao et al. [165] successfully prepared Ag-coated ZnO nanoparticles using a green synthesis method based on *Tribulus terrestris* leaf extract. The Ag nanoparticles ranged from 6 to 10 nm in diameter. The results showed the superior ethanol sensing properties of the synthesized Ag-coated ZnO nanoparticles at room temperature: the resistance values of pure ZnO sensor decreased continuously from 2.5 MΩ to 1.25 MΩ following two straight lines with different slopes, while the resistance values of Ag-coated ZnO sensor dropped down linearly from 0.5 MΩ to 0.24 MΩ in the range 0–20 ppm. Goutham et al. [166] synthesized ZnO nanoparticles applying two approaches, i.e., green synthesis (from *Aloe vera* extract and zinc nitrate) and chemical synthesis (solution combustion method with zinc nitrate and glycine) and compared their sensing performance towards various concentrations of LPG gas at different working temperatures. In addition, they investigated the structure of synthesized particles by field emission scanning electron microscope (FE-SEM). They found that the green synthesized ZnO nanoparticles (Figure 17a,b) were randomly distributed in spherical morphology while chemically synthesized zinc oxide powder was spherical in nature (Figure 17c,d) with a particles size of 50–60 nm for both prepared ZnO. The size of synthesized particles improved the physical adsorption of a high amount of gas analyte molecules on the surface, which caused changes in the electric resistance of the sensing film.

**Figure 18:**
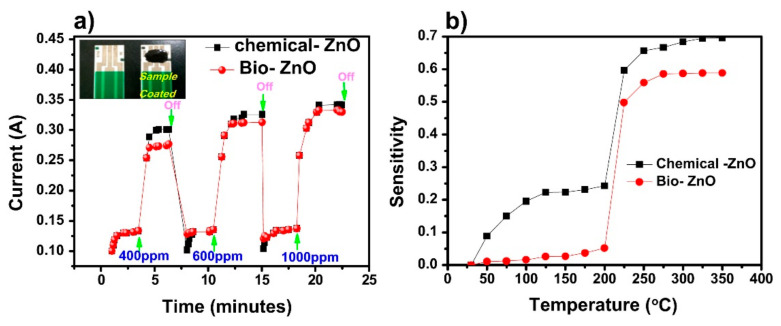
(**a**) Sensing response of chemical-ZnO and bio-ZnO devices towards various concentrations of LPG; (**b**) the effect of temperature on the sensitivity of the chemical- and bio-ZnO devices towards 1000 ppm of LPG. Reprinted from Ref. [166] with permission of Elsevier Ltd., 2016.

Furthermore, the results demonstrated that the green synthesized ZnO showed maximum current change at 1000 ppm of LPG. As shown in Figure 18a, with increasing the concentration of LPG up to 1000 ppm, the sensitivity and response of the sensor increased with respect to the working temperature. Moreover, the effect of temperature on the sensitivity of the prepared sensors was evaluated in the range from room temperature to 350 °C (Figure 18b). It can be seen that both the chemical-ZnO and bio-ZnO sensors showed the maximum sensitivity at 250 °C for 1000 ppm of LPG. The authors reported that the chemically synthesized ZnO sensor presented similar sensing characteristics as the biosynthesized ZnO. Although, the biosynthesized ZnO sensor showed a lower sensitivity compared to the chemically synthesized one (Figure 18b).

A list of studies dealing with ZnO NPs has been summarized in Table 6.

### 6.2. Titanium Oxide Nanoparticles (TiO_2_ NPs)

Titanium dioxide, or titania, is one of the most widely synthesized metal oxide nanomaterials due to its excellent and versatile features. Titanium dioxide is commonly applied in medicine, water, and air purification as a photocatalyst due to its potential oxidation strength, long-term stability, and non-toxicity [169,170]. The TiO_2_ NPs have also been utilized in high-power Li-ion batteries [171] and in sensing applications, for example, for the detection of volatile chemicals and gases, as well as chemical oxygen demand sensors and biosensors for biological substances.

Plant extracts have been reported as effective sources in the synthesis of TiO_2_ NPs with antimicrobial potential [172], and solar cell ability [173] or photocatalytic activity [174]. Titanium dioxide NPs with different structures and sizes have also been achieved in recent years via microbes, and through fungal-mediated synthesis; TiO_2_ NPs with a 28–54 nm particle size were synthesized through *Aeromonas hydrophila* (bacteria) in ref. [112]. A study performed by Rajakumar et al. evidenced that fungi were beneficial for synthesizing TiO_2_ NPs due to their biodegradable nature [175]. Researchers have also obtained spherical TiO_2_ NPs using *Planomicrobium* sp. and *Aspergillus niger* with the size of 73–100 nm [176,177].

However, the synthesis of NPs via plant-mediation leads to more stable products than with microbe-mediated synthesis [178]. In addition, leaves are richer in metabolites, thus, they are more widely used to produce extract [53]. In 2022, Sagadevan et al. [53] summarized biosynthesized TiO_2_ NPs through plant extract and microbes (fungi and bacteria): clustered or polydispersed, spherical, quasi-spherical, oval, or tetragonal particles ranging from 2 to 150 nm can be obtained in function of the selected precursors. For example, tetragonal TiO_2_ NPs with a particle size of 47 nm were synthesized using leaf extracts of *Ledebouria revoluta* (African hyacinth). While, spherical TiO_2_ NPs were obtained through *Hibiscus rosa-sinensis* (flower), *Acanthophyllum laxiusculum* (root), and *Cicer arietinum* L. (seed) with the particle size in the range of 7–25 nm [171,179,180].

### 6.3. Cerium Oxide Nanoparticles (CeO_2_ NPs)

Cerium oxide is a rare earth n-type semiconducting material with high crystallographic stability (up to its melting point, 2700 °C), high exciton binding energy, and wide bandgap energy from 3.0 to 3.2 eV, and UV wavelength at around 370 nm [98]. Because it can switch between its oxidation states, it can be widely used as a catalyst, gas sensor, energy storage, cosmetics, etc. [181]. Cerium oxide is also gaining considerable attention from researchers as a sensor for the detection of humidity and various gases such as NH_3_ [182], H_2_ [183], CH_2_O [184], and NO_2_ [185]. Extensive efforts have been focused on the green synthesis of CeO_2_ NPs using an eco-friendly, cost-effectiveness, time-saving, and high-yield approach without producing toxic residues. Cerium oxide nanoparticles have been green synthesized using alga, extracellular compounds of fungi, and plant extracts (leaves, stem, seeds, etc.). For example, CeO_2_ NPs were produced with an average particle size of 22 nm using an aqueous extract of *Acorus calamus* and cerium nitrate in ref. [186].

**Figure 19:**
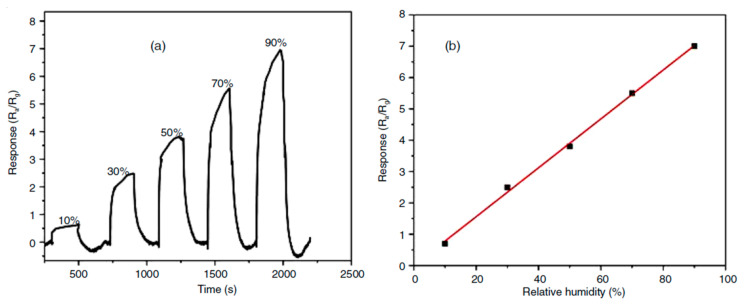
(**a**) Ce_2_O gas sensing performance towards humidity (10–90%); (**b**) linear fitting of response. Reprinted from Ref. [181].

Porous cerium oxide nanoparticles were first synthesized using *Mimosa pudica* plant leaf extract and cerium nitrate in a study conducted by Kulkarni et al. [151] to detect humidity. They found that the resistance of the CeO_2_ sensor decreased when exposed to 10–90% of relative humidity (RH). The response of the CeO_2_ sensor is shown in Figure 19a. The response time was reported from 33 s to 12 s against 10% RH and 90% RH, respectively. Moreover, recovery time varied between 15 s for 10% RH and 59 s for 90% RH. The linear fitted response regarding humidity is illustrated in Figure 19b.

### 6.4. Iron Oxide Nanoparticles (Fe_3_O_4_ NPs)

In recent years, the environment has been polluted by fertilizers and pesticides [187,188]. Thus, magnetic nanoparticles attracted tremendous regard to the restoration of the environment, including cleaning the atmosphere, soil, sedimentary rocks, groundwater, and surface water [5]. Due to their high specific surface area, they act as “super-absorbents” in the case of many contaminants to convert them into non-toxic compounds. The most common natural iron oxide is magnetite Fe_3_O_4_ with an inverse spinel structure [43]. The unique features of iron oxide nanoparticles make them widely utilized in various fields, namely: superparamagnetic, magnetic resonance imaging, catalysis, gas sensing, optics, water purification, lithium-ion batteries, biomedical applications, etc. [5,44].

Magnetite nanoparticles (Fe_3_O_4_ NPs) were biologically synthesized using brown seaweed, *Carica papaya*, *Ocimum sanctum*, monocotyledons, and dicotyledons plants [3]. Extensive efforts have been focused on fabricating Fe_3_O_4_@ZnO core-shell nanoparticles (45–64 nm in size) using *Azadirachta indica* (neem) leaf extracts (AI leaves) with ferrous sulfate, ferric nitrate nonahydrate, zinc chloride and sodium hydroxide in ref. [189,190]. Different biosynthesis based on starch (a hydrophilic polymer that is an effective dispersing and stabilizing agent), sodium alginate, ascorbic acid (Vitamin C; to reduce transition metal salts), amino-acids (L-lysine, L-glutamic acid, L-glutamine, L-arginine, and L-cysteine), synthetic tannic and gallic acid and co-precipitation or hydrothermal synthesis were reviewed in ref. [159]. Lu et al. [191] prepared spherical Fe_3_O_4_ nanoparticles with an average size of about 12.5 nm using D-glucose as the reducing agent and gluconic acid (oxidized glucose) as a stabilizer and dispersant, respectively. Iron reducing bacteria are also commonly used to synthesize iron nanomaterials, as well as fungi, algae, plant extracts (green tea, containing polyphenols, eucalyptus, carob leaf, mango, rose, oregano, curry leaves, and others), fruit extracts, and seed extracts (*Syzygium cumini*) [159]. Moreover, a study by Salehzadeh et al. [192] was conducted on the synthesis of Ag/Fe_3_O_4_ using *Spirulina platensis* algae extraction.

**Figure 20:**
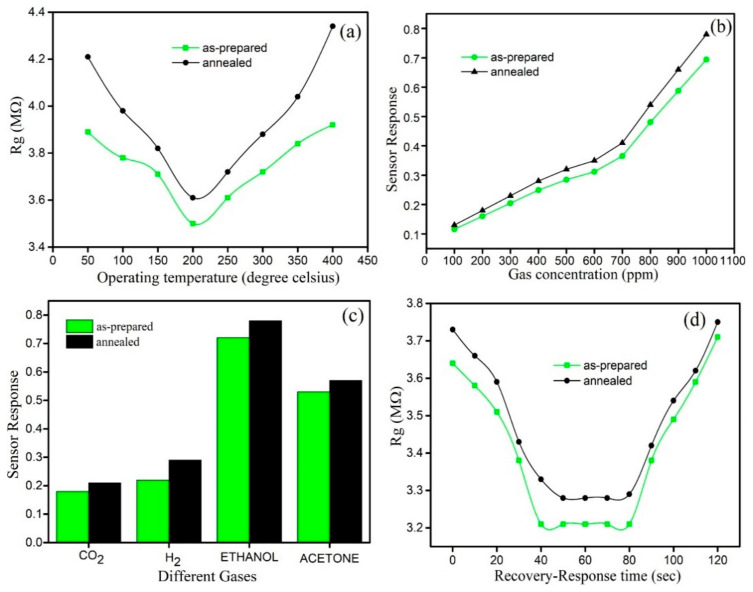
Gas sensor performance for synthesized (Fe_3_O_4_ NPs), (**a**) The effect of temperature on the sensor resistance of the as-prepared and annealed samples_;_ (**b**) the sensor response towards various ethanol concentrations at 200 °C; (**c**) the sensor response of the samples under 1000 ppm of different gases; (**d**) the response and recovery time of samples to 1000 ppm of ethanol at 250  °C. Reprinted from Ref. [193] with permission of Elsevier Ltd., 2022.

Ananthi et al. [193] synthesized iron oxide nanoparticles (Fe_3_O_4_) using ferric nitrate nonahydrate and a natural tannic acid (extracted from green tea, used as a capping agent) assisted combustion approach followed by calcination. To determine the working temperature, the resistance of the sensor was measured at various temperatures from 50 to 400 °C towards 1000 ppm of ethanol. As shown (Figure 20a), by increasing the temperature up to 400 °C, there was a steady decrease in the sensor’s resistance, which reached a minimum value at approximately 200 °C, considered the operating temperature. Figure 20b shows the sensor response towards various ethanol concentrations at 200 °C for as-prepared and 350 °C annealed samples. Interestingly, both specimens demonstrated a similar sensor response variation in the presence of ethanol. The maximum sensor response was obtained in the presence of 1000 ppm of ethanol, and the sensor response reached 0.72 and 0.68 for the annealed and as-prepared specimens, respectively. Moreover, the authors investigated the sensor response of the samples toward 1000 ppm of four different gases, including ethanol, acetone, H_2_, and CO, at 250  °C (Figure 20c). Concerning the other gases, the specimens exhibited a strong sensor response towards ethanol and acetone, although a higher sensor response was obtained for ethanol. The response time and recovery time reported were about (48 s and 46 s) and (32 s and 30 s) for the as-prepared and annealed samples, respectively, for 1000 ppm of ethanol at 250  °C (Figure 20d).

**Figure 21:**
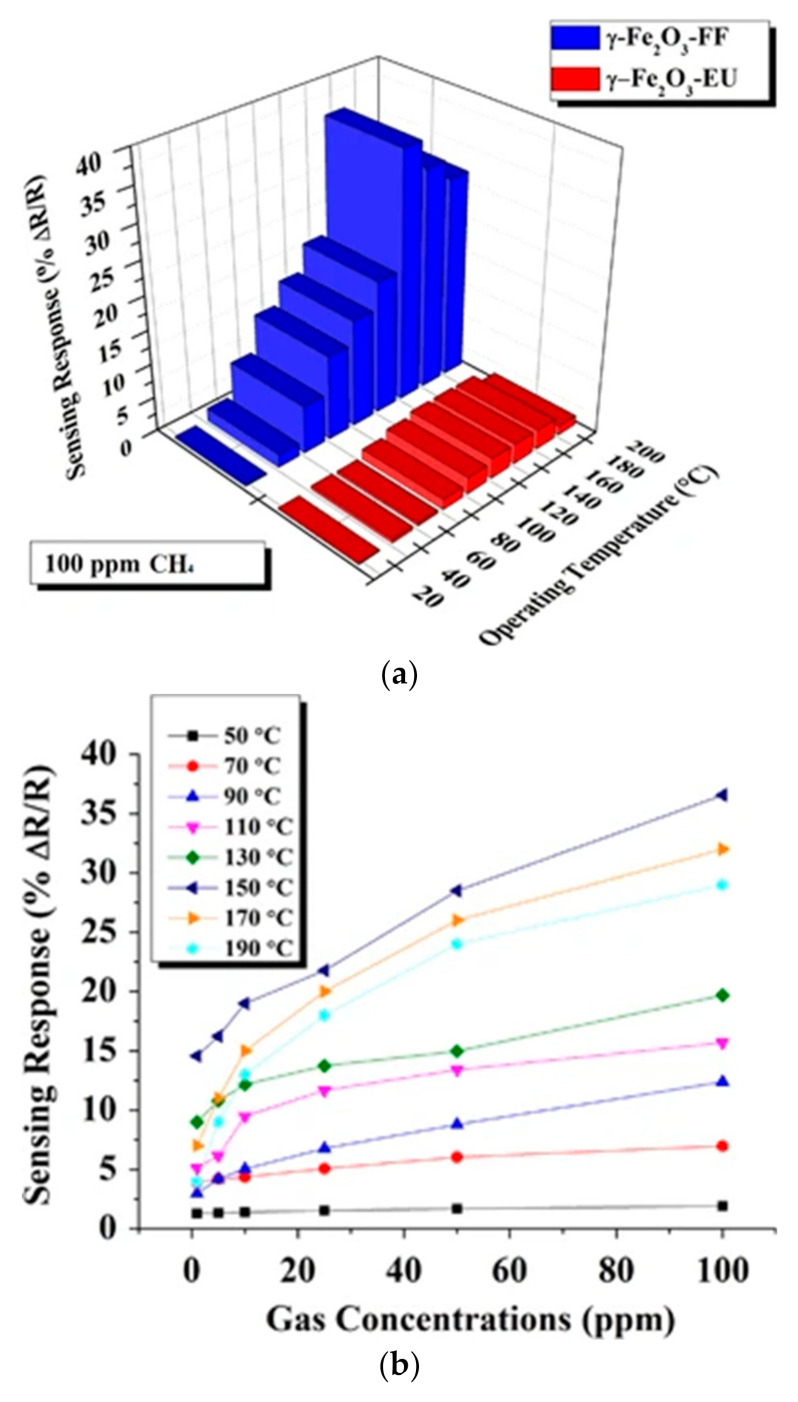
The gas response of the γ-Fe_2_O_3_–EU and γ-Fe_2_O_3_–FF NPs: (**a**) towards 100 ppm of CH_4_ as a function of working temperature (25–190 °C); (**b**) responses of the γ-Fe_2_O_3_–FF versus gas concentrations. Reprinted from Ref. [104] with permission of Springer Science Business Media, LLC, 2017.

Karaduman et al. [104] developed trace-level methane gas detection using γ-Fe_2_O_3_ nanoparticles. They used FeCl_2_ and Fe(NO_3_)_3_ (in a stoichiometric ratio of 2:1 (Fe^3+^/Fe^2+^)) extracts of the *Ficus carica* and *Euphorbia amygdaloides* plants labeled as γ-Fe_2_O_3_–FF NPs and γ-Fe_2_O_3_–EU, respectively. The response of synthesized sensors was evaluated for 100 ppm of methane in the range of temperature from 25 to 190 °C (Figure 13). By increasing the temperature, the response of γ-Fe_2_O_3_–EU was almost constant, while γ-Fe_2_O_3_–FF presented the highest response (36 at 150 °C, Figure 21a). Moreover, the sensor’s response was enhanced with increased gas concentration (Figure 21b). Based on the experimental results, γ-Fe_2_O_3_–FF presented an acceptable response above 50 °C towards all methane concentrations. However, the maximum response was obtained at 150 °C, considered the optimum operating temperature to detect CH_4_ with rapid sensing characteristics. In contrast, γ-Fe_2_O_3_–EU showed a 15% response versus 1 ppm of methane at 150 °C. Therefore, γ-Fe_2_O_3_–FF demonstrated a higher response, better selectivity, and shorter response/recovery time with respect to γ-Fe_2_O_3_–EU.

Cao et al. [194] prepared perovskite iron yttrium oxide (FeYO_3_) microspheres from iron nitrate nonahydrate, yttrium (III) nitrate hexahydrate, citric acid monohydrate, C_19_H_42_BrN (CTAB) and the inner white part of orange peels using hydrothermal ‘green synthesis’ to detect ethanol. The sensing characteristics of the FeYO_3_ sensor were measured in the presence of 25 ppm of ethanol versus various testing temperatures (290–390 °C). By increasing the temperature, the sensor response gently increased and reached a maximum value of 7.32 at 330 °C with the response and recovery times of 30 s and 56 s, respectively. Moreover, the authors measured the sensor response at 330 °C under ethanol concentrations from 1 to 200 ppm. It was found that the response of the FeYO_3_ sensor sharply increased by increasing the concentration of ethanol up to 70 ppm. In addition, the synthesized sensor possessed a low detection limit of 1 ppm of ethanol with a response value of 2.38. The selectivity to ethanol at 330 °C was reasonably good (the sensor response was about 7.2 under 25 ppm ethanol while it was equal to ca. 6 when exposed to 25 ppm methanol). Bangale et al. [195] successfully fabricated nanocrystalline CdFe_2_O_4_ through a combustion technique, using citric acid as fuel. The precursors were cadmium nitrate hexahydrate and iron nitrate hexahydrate. They reported a response as high as 59.23% when the sensor was exposed to 50–200 ppm of ethanol. In addition, the response time and recovery time were approximately 40 s and 50 s, respectively. They revealed that the CdFe_2_O_4_ sensor exhibited high sensitivity and rapid response/recovery to ethanol at 350 °C. Other examples of iron oxide nanoparticles obtained via the green chemistry approach are summarized in Table 7.

### 6.5. Indium Oxide Nanoparticles (In_2_O_3_ NPs)

Indium oxide is an n-type metal oxide semiconductor having a direct bandgap (Eg = 3.5–3.7 eV). Due to great electrical, optical, physical, and chemical features, In_2_O_3_ has vast applications as a heterogeneous catalyst in solar cells, optoelectronic devices, photocatalysts, and transparent conducting oxide [196,197]. Moreover, In_2_O_3_ micro/nanostructures have been extensively used for gas sensing owing to their excellent selectivity, short response/recovery time, and high response. Various chemical and physical techniques are applied to synthesize indium oxide, such as thermal evaporation, hydrothermal, spray pyrolysis, sol-gel, etc. [197,198]. Synthesized indium oxide nanostructures exist in various morphologies such as nanorods, nanocubes, nanosheets, nanobelts, nanoparticles, nanofibers, nanowires, and flower-like. There are many reports on the use of In_2_O_3_ in gas sensor applications to detect oxidizing and reducing gases such as CO_2_, H_2_S, O_3_, ethanol, NH_3,_ NO_2,_ etc. [197].

**Figure 22:**
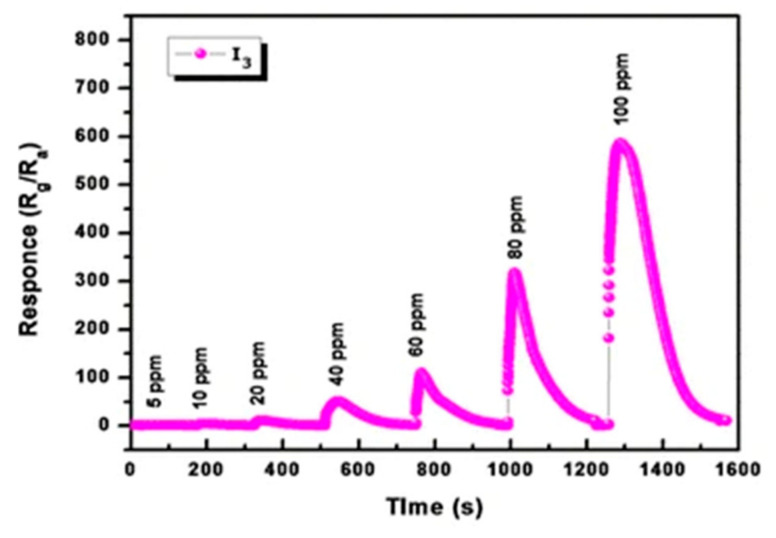
Sensor response of In_2_O_3_ film at various concentrations of NO_2_. Reprinted from Ref. [197] with permission of Springer Science Business Media, LLC, 2018.

Indium oxide bricks were prepared by a hydrothermal route from indium chloride and L-alanine as a reducing agent in a study conducted by Pawar et al. [197]. These authors studied the gas sensing response of screen-printed In_2_O_3_ films at different working temperatures (50–200 °C) towards various concentrations of NO_2_ in the range from 5 to 100 ppm. In the beginning, the response of In_2_O_3_ thin film dramatically increased from 12 to 140 at 50 °C and 100 °C, respectively, under 60 ppm of NO_2_. However, by increasing the temperature to 200 °C, the sensor’s response decreased to 72 and 73 at 150 °C and 200 °C, respectively. Therefore, the optimum working temperature for In_2_O_3_ film was reported to be approximately 100 °C. In addition, it was observed that when increasing NO_2_ concentration up to 100 ppm, the sensor response increased too (Figure 22).

**Figure 23:**
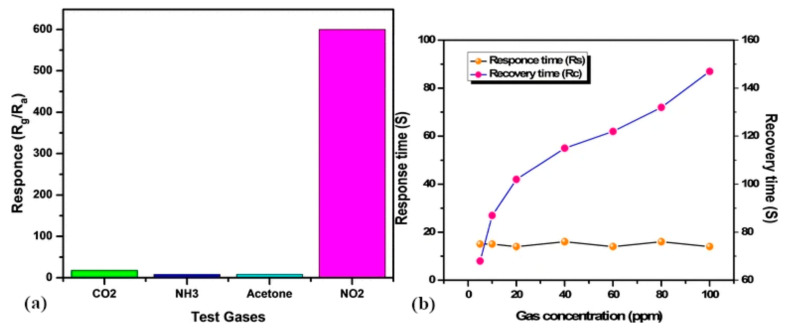
(**a**) The selectivity of indium oxide thin film sensor for 100 ppm of various gases at 100 °C; (**b**) response/recovery time of indium oxide towards various concentrations of NO_2_ gas. Reprinted from Ref. [197] with permission of Springer Science Business Media, LLC, 2018.

The selectivity of screen-printed In_2_O_3_ films was additionally assessed at the working temperature of 100 °C towards 100 ppm of CO_2_, NH_3_, NO_2,_ and acetone (Figure 23a). It was found that the fabricated sensor showed the highest selectivity towards NO_2_. Quite fast response and recovery times are observed for different concentrations of NO_2_ gas (Figure 23b), which refers to rapid adsorption and desorption of test gas on the surface of In_2_O_3_ film. The list of reported studies is presented in Table 8.

### 6.6. Copper Oxide Nanoparticles (CuO NPs)

Copper oxide is a p-type semiconductor with a narrow bandgap (1.2 eV). It has attracted particular attention due to its low-cost, safety, high stability, great catalytic activity, and adsorption performance [200,201]. Copper oxide NPs have numerous applications, including gas sensing, catalysis, batteries, food preservation, superconductor, dye degradation, waste treatment, solar energy transformation, agriculture, cosmetics, biomedical, environmental science, etc. [202]. Mainly, CuO nanoparticles are used as a gas sensing material to detect various gases such as NO_2_, acetone, ethanol, H_2,_ etc. [200,203,204]. Findings have also proved that copper oxide thin films can be applied as gas sensors to detect other compounds such as carbon oxides, hydrogen sulfate, and ammonia with great sensor performance (high sensitivity, selectivity, and stability). Moreover, these sensors can operate at ambient temperature with long-term stability [205].

In the literature, several procedures are reported for the synthesis of CuO NPs, such as sol-gel, sonochemical, electrochemical, reverse micelle, hydrothermal, precipitation, thermal decomposition, laser ablation, microwave, and alkoxide based methods. Nevertheless, these chemical and physical methods suffer many disadvantages, including high energy consumption, complex purification of NPs, producing a large amount of waste, as well as the need to use dangerous chemical materials, flammable organic solvents, and expensive instruments. To overcome these challenges, it is necessary to develop the green synthesis of CuO nanomaterials by fast and energy-efficient techniques based on non-toxic raw materials [206]. Table 9 presents a comparison between various methods for synthesizing CuO NPs.

The investigations on the green synthesis of Cu NPs and CuO NPs have increased with respect to physical and chemical methods. The CuO NPs were prepared by Dey et al. [149] using cupric sulfate and the aqueous extract of AI leaves. The green synthesis of copper oxide from different roots, husk, fruit, leaves, and aerial parts of plants and copper sulfate, acetate, nitrate, or chloride is reviewed in [207,208].

**Figure 24:**
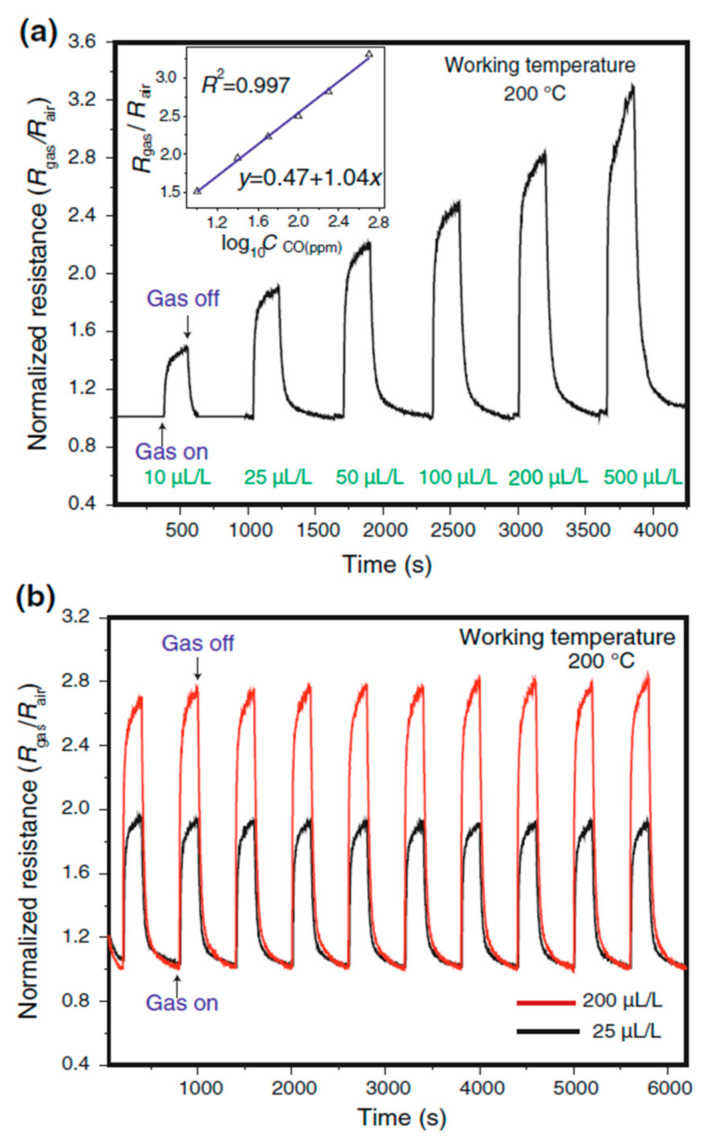
(**a**) Response of Cu_2_O/Au/CuO sensor towards different concentrations of CO gas at 200 °C; the inset figure is the plot of Rgas/Rair as a function of the CO concentration; (**b**) Reproducibility of the sensor on (10 cycles) to 25 against 200 µL/L CO. Reprinted from Ref. [209] with permission of Springer Science Business Media, LLC, 2013.

Hollow Cu_2_O/Au/CuO hierarchical nanostructures were obtained using NaOH solution added into an aqueous solution of CuCl_2_·2H_2_O at 55 °C, followed by ascorbic acid solution in a study conducted by Liu et al. [209]. The sensing materials were used as CO gas sensors. They reported that the unique hierarchical structure of synthesized composite materials with a high specific surface area makes them a promising candidate for high-performance CO gas sensors. It was observed that the catalytic activity of gold leads to structure revolution and promotion of CO gas sensing performance. They measured the sensor response against 10, 25, 50, 100, 200, and 500 µL/L of CO gas at 200 °C (Figure 24a). The increase in CO concentration caused a linear increase in the sensor response. The synthesized Cu_2_O/Au/CuO sensor presented a response of 1.5 even in the presence of a low concentration of CO (10 µL/L). Figure 24b illustrated the reproducible response and recovery signals of the sensor towards low (25 µL/L) and high concentrations (200 µL/L) of sensing gas which indicated excellent long-term stability of the Cu_2_O/Au/CuO sensor. Nevertheless, they highlighted that the Cu_2_O nanocubes do not show any detectable response without the addition of Au under the same working condition. The sensor response in function of the gas concentration was linear (inset of Figure 24a) with a sensitivity of 1.04 ppm^−1^ (the slope of the calibration curve).

### 6.7. Nickel Oxide Nanoparticles (NiO NPs)

Recently, nickel oxide nanoparticles have attracted much research interest due to their unique chemical stability, electrocatalysis, electron transfer capability, and super-capacitive characteristics [210,211]. It is a chemically stable p-type semiconductor having a wide bandgap of 3.6–4.0 eV and high electro-optical efficiency [212]. The NiO NPs are extensively applied in various fields, including antiferromagnetic materials, gas sensing, solar and fuel cells, lithium-ion batteries, electrochemical supercapacitors, magnetic and antibacterial materials, and photocatalytic agents [213]. Nickel oxide nanoparticles have been successfully produced using various chemical and physical methodologies such as anodic arc plasma, sol-gel, precipitation, solvothermal synthesis, microwaves, and thermal decomposition. Nevertheless, these conventional methods are not eco-friendly, produce hazardous by-products, and require high energy. The biosynthesis approach has attracted tremendous regard for producing NiO NPs using plants and various microorganisms [213].

**Figure 25:**
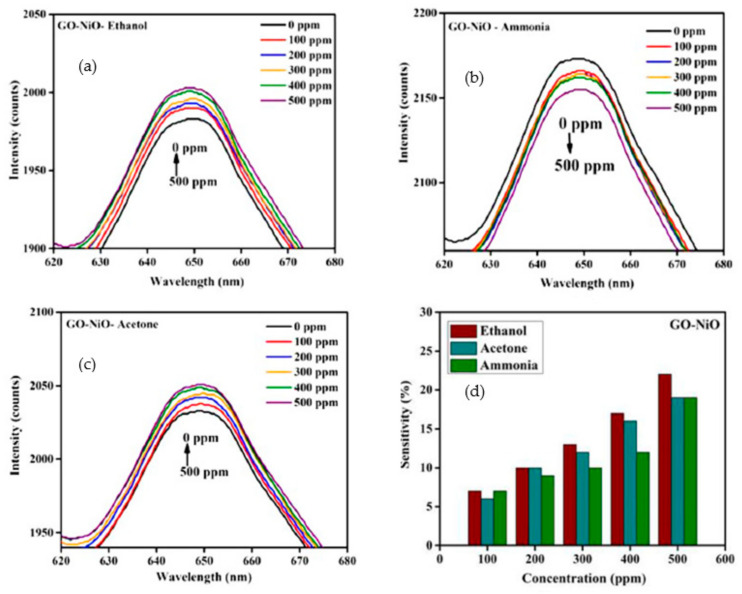
Spectral response of GO-NiO nanocomposite sensing as modified fiber optic sensor towards (**a**) Ethanol; (**b**) Ammonia; (**c**) Acetone; (**d**) Sensitivity (%) of GO-NiO sensor towards (0–500 ppm) concentrations of test gases at room temperature. Reprinted from Ref. [215] with permission of Springer Science Business Media, LLC, 2022.

Kennedy et al. [214] used biosynthesis methods to prepare doped nanocrystalline NiO as a humidity sensor (derived from the *Hygrophila spinosa* plant seeds, HST, nickel nitrate hexahydrate, and polyvinyl alcohol). After air drying, a PMMA optical fiber was then mechanically etched and dip-coated with GO-NiO nanocomposites and inserted into the glass chamber. It was observed that the 0.5% HST-NiO sample had good linearity and reproducibility. Moreover, this sample exhibited response and recovery times of 210 ± 5 s and 232 ± 4 s, respectively, with respect to the pure NiO sample. In 2022, a study conducted by Kavitha et al. [215] resulted in the synthesis of NiO NPs using *Tribulus terrestris* leaf extract and nickel nitrate hexahydrate. These nanoparticles were mixed with graphene oxide nanosheets (GO) to obtain GO-NiO nanocomposites. The sensing ability of GO-NiO towards volatile organic compounds (VOCs) was assessed for the first time by a fiber-optic gas sensor at room temperature. Figure 25a–c demonstrates the spectral response of the GO-NiO sensor towards various concentrations (0–500 ppm) of ammonia, acetone, and ethanol.

The intensity of the spectral response changed with all test gas concentrations, although there was no peak shift observed. Moreover, the authors reported a superior spectral response for ethanol compared to acetone and ammonia, which displays an increase in the intensity of ethanol with increasing concentration. The sensor response (%) of GO-NiO nanocomposite for 0–500 ppm of ammonia, acetone, and ethanol is exhibited in Figure 25d.

The sensor response reached a maximum value of 22% for 500 ppm of ethanol at room temperature and was higher than for other VOCs gases. This result was attributed to the synergic effect of GO, their small particle sizes and narrow band gap. The fit of the curves displaying the sensor response in function of the gas concentration showed a linear regression in spectral response for ethanol and acetone gases with positive slope values of 4.085 and 3.607 respectively. On the contrary, with ammonia, the spectral response decreased when increasing the gas concentration, probably because the effective refractive index of the fiber was influenced by an increased leakage of light. Table 10 reports studies on nickel oxide nanoparticles as gas sensing materials.

### 6.8. Tin Oxide Nanoparticles (SnO_2_ NPs)

Tin oxide nanoparticles have gained remarkable attention as an n-type semiconductor with a wide bandgap of 3.6 to 3.8 eV, strong thermal stability (up to 500 °C), strong chemical and physical interactions with the adsorbed species and a high degree of transparency in the visible spectrum. These unique properties make it a promising candidate in various applications, including gas sensing, lithium batteries, transparent conducting electrodes, catalysis, energy storage, glass coatings, medicine, and environmental remediation [216,217]. Among these applications, extensive investigations have been focused on the application of SnO_2_ in gas sensing due to its high specific surface area (SSA), high chemical stability, low cost, easy synthesis, good light transmission and high electron mobility (160 cm^2^⋅V/s), low electrical resistance, and low density for the detection of exhaust gases such as CO, NO, NO_2_ and H_2_S, as well as volatile organic compounds like C_2_H_5_OH. The gas sensing properties of SnO_2_-based devices are greatly influenced by their morphologies, such as nanoparticles, nanorods or nanowires, nanosheets, nanoflowers, nanotubes, mesoporous hollow spheres, and hierarchical nanostructures. However, three-dimensional SnO_2_ hierarchical nanostructures are considered effective gas sensing materials due to their large SSA, porous nanostructure, and controlled size [130,218,219].

**Figure 26:**
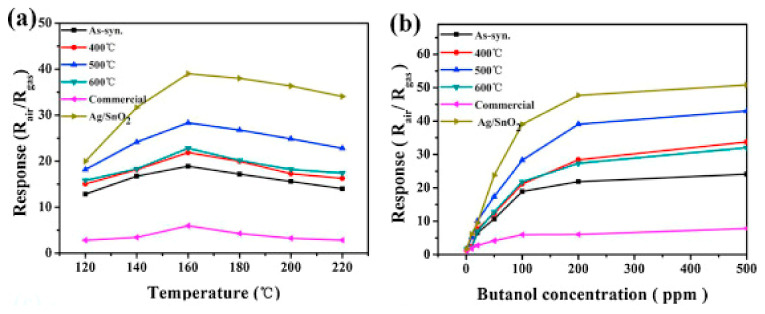
A comparison between sensor response SnO_2_ NPs: (**a**) 100 ppm butanol at various working temperatures; (**b**) response of sensor at 160 °C towards various concentrations of butanol (5–500 ppm). Reprinted from Ref. [220] with permission of Elsevier B.V., 2015.

A flower-like tin oxide hierarchical nanostructure was first synthesized from tetrapropylammonium hydroxide and SnSO_4_ through a hydrothermal technique and an impregnation method with ascorbic acid, and AgNO_3_ was then used to produce Ag/SnO_2_ nanostructures [220]. As shown in Figure 26a, the sensitivity of SnO_2_ NPs annealed at 500 °C was much higher than commercial SnO_2_ and samples annealed at 400 °C and 600 °C. The response of flower-like SnO_2_ NPs under 100 ppm of butanol gradually increased from 120–160 °C and reached a max response of 28.3 at 160 °C. However, by increasing the temperature up to 220 °C, the sensor response decreased. By incorporating the Ag NPs into SnO_2_, the sensor’s sensitivity toward butanol increased to 39.9 at 160 °C, which was much higher than pure SnO_2_ NPs. Therefore, 160 °C was considered the working temperature. Moreover, the sensor’s sensitivity toward various concentrations of butanol (1–500 ppm) was evaluated at this operating temperature. The results showed that tin oxide annealed at 500 °C exhibited higher sensitivity than those samples annealed at 400 °C and 600 °C towards different butanol concentrations (Figure 26b). In addition, the sensor response of SnO_2_ NPs significantly increased to the maximum by incorporating Ag.

**Figure 27:**
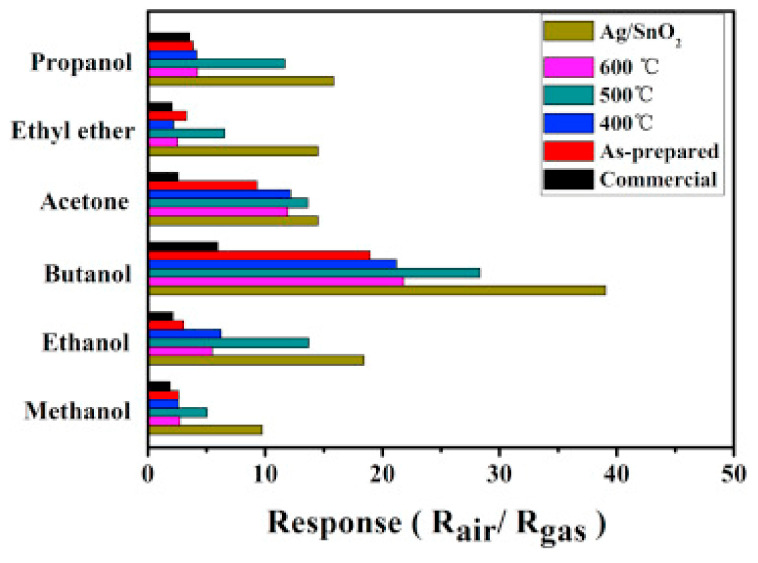
Sensor response of SnO_2_ NPs-based sensors towards 100 ppm of different gases at the optimal working temperature. Reprinted from Ref. [220] with permission of Elsevier B.V., 2015.

The sensor’s selectivity under 100 ppm of test gases was measured towards methanol, propanol, acetone, ethanol, ethyl ether, and butanol (Figure 27). The sensitivity to all gases was lower than that toward butanol. Moreover, pure SnO_2_ and Ag/SnO_2_ sensors demonstrated better sensor response to butanol at an operating temperature of 160 °C compared with commercial SnO_2_ NPs sensors.

**Figure 28:**
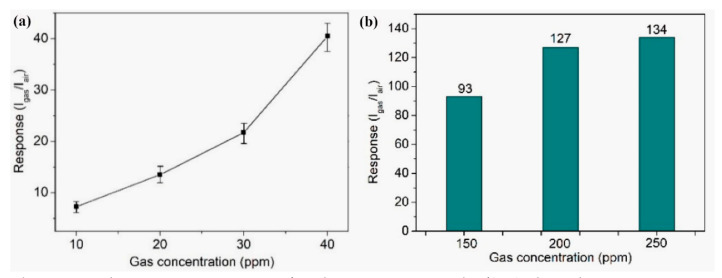
The sensing response of carbonaceous particles/SnO_2_-based gas sensor towards ethanol: (**a**) at concentrations 10, 20, 30, and 40; (**b**) response toward 150, 200, and 250 ppm of ethanol. Reprinted from Ref. [221].

Ding et al. [221] fabricated the first carbonaceous particles (1 μm in diameter) by hydrothermal synthesis from wheat or corn. Then, they used them as a sacrificial template for preparing hollow SnO_2_ nanosphere from tin(IV) chloride. The prepared sensors were able to detect ethanol at various concentrations. The authors reported a high sensing performance of 134 towards 250 ppm of ethanol at 200 °C (Figure 28).

**Figure 29:**
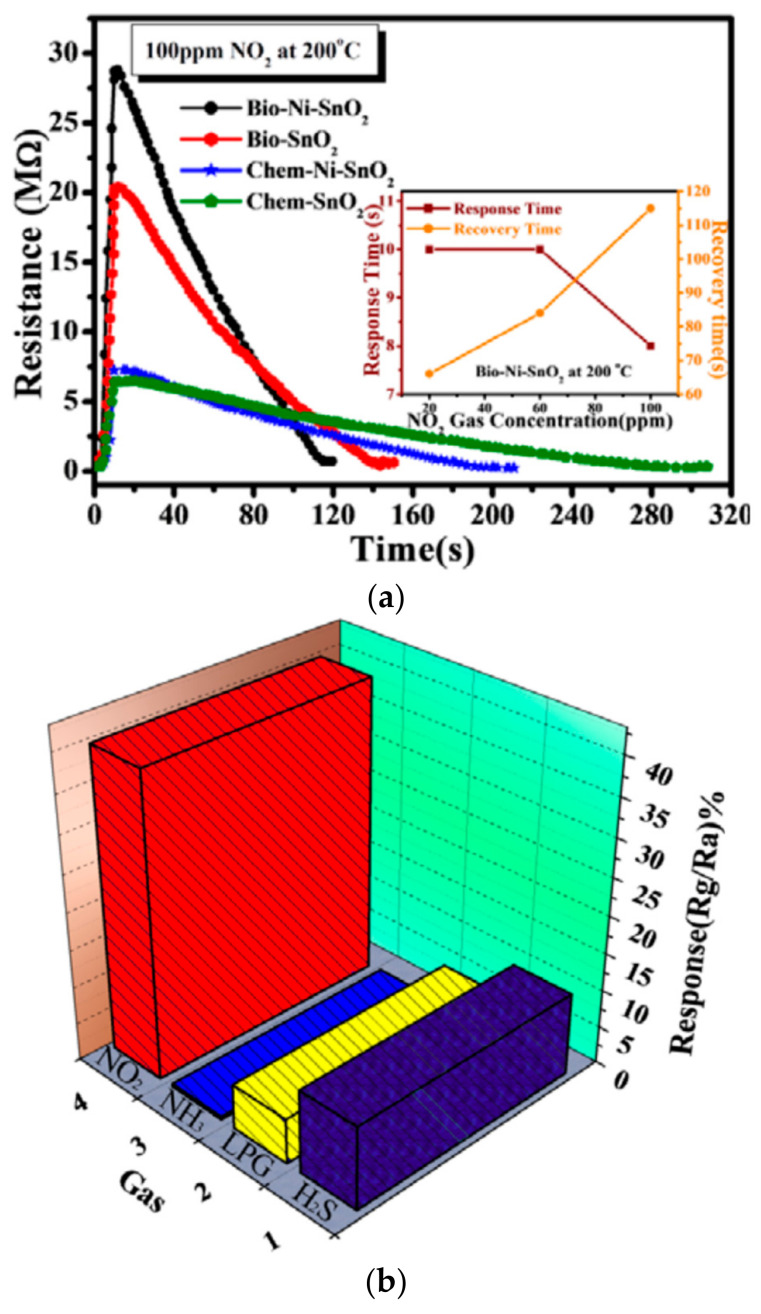
(**a**) Gas sensitivity towards various concentrations of NO_2_ for chemically and green synthesized SnO_2_ and Ni-doped SnO_2_ nanoparticles thin film; Inset illustrates the response and recovery time with variation in the concentration of NO_2_ for both specimens; (**b**) Gas selectivity for Ni-doped SnO_2_ sensor towards 100 ppm concentration of H_2_S, LPG, NH_3,_ and NO_2_. Reprinted from Ref. [223] with permission of the Royal Society of Chemistry, 2011.

A study conducted by Prajabati et al. [222] described the synthesis of SnO_2_/Carbon Quantum Dots (CQDs) by a hydrothermal method using grapefruit juice. They investigated the sensor response toward 1000 ppm of carbon monoxide at various operating temperatures. The synthesized SnO_2_/CQDs showed the highest performance at 275 °C. Incorporating CQDs into SnO_2_ has an essential effect on the gas sensing response and selectivity. Gattu et al. [223] compared the gas sensing performance of both chemically (sol-gel method) and biosynthesized pure SnO_2_ and Ni-doped SnO_2_ made from Bengal gram beans (*Cicer arietinum*) extract for detection of NO_2_ gas at 200 °C (Figure 29a). In the presence of Ni, the sensor response increased for both specimens, which can be attributed to the reduced particle size when adding Ni and increased specific surface area for better absorption of NO_2_ gas.

Nevertheless, the response was relatively lower for chemically synthesized Ni-doped SnO_2_ with respect to biosynthesized Ni-doped SnO_2_. In the case of biosynthesized Ni-SnO_2_, there was a decrease in response time and an increase in recovery time with the concentration of NO_2_ gas. Furthermore, their results showed that Ni-doped SnO_2_ has excellent selectivity for detecting NO_2_ compared to H_2_S, LPG, and NH_3_ gases (Figure 29b).

**Figure 30:**
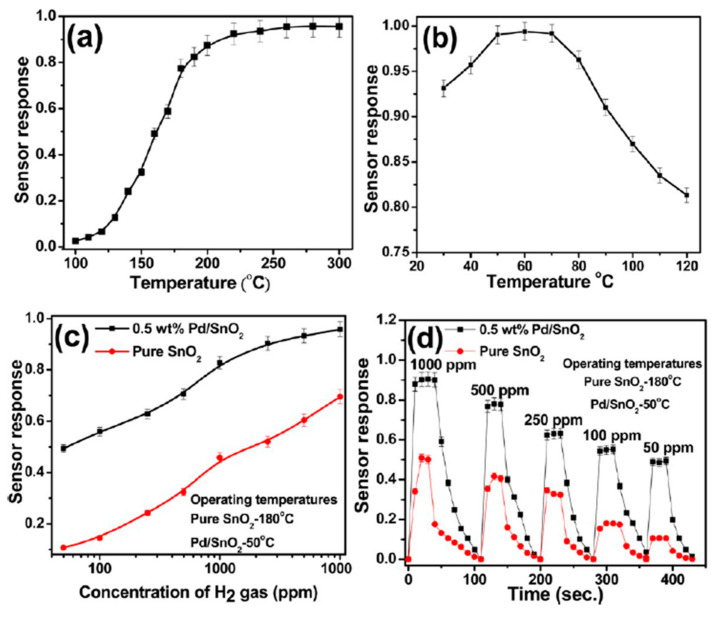
(**a**) Sensor response of pure SnO_2_ at various working temperatures to 1% H_2_ gas; (**b**) sensor response of Pd/SnO_2_ at different temperatures to 1% H_2_ gas; (**c**) sensor response of pure SnO_2_ and Pd/SnO_2_ toward various concentrations of H_2_ gas at operating temperatures of 180 and 50 °C, respectively; (**d**) Sensor response of pure SnO_2_ and Pd/SnO_2_ as a function of time at 180 and 50 °C. reprinted from Ref. [219] with permission of the American Chemical Society, 2012.

In another study, Gattu et al. [224] investigated the sensing performance of Au-doped SnO_2_ thin film to NO_2_ gas at 200 °C operating temperature. Gold doped SnO_2_ nanoparticles were manufactured from remnant water collected from soaked Bengal gram beans (*Cicer arietinum*) extract. The authors found that Au/SnO_2_ showed a gas response of about 30% to 100 ppm of NO_2_. Moreover, the biosynthesized sensor presented excellent selectivity to NO_2_ gas compared to other gases such as H_2_S, LPG, and NH_3_. Prajapati et al. [222] evaluated the gas sensing properties of SnO_2_/Carbon Quantum Dots (CQDs) nanocomposite synthesized by the hydrothermal method. The maximum response towards 1000 ppm of carbon monoxide was reported at 275 °C. Manjula et al. [219] prepared biosynthesized pure SnO_2_ nanosphere and Pd/SnO_2_ from sodium stannate trihydrate precursor and glucose as structure modifying agents to detect hydrogen gas. It was observed that the pure SnO_2_ sensor response reached saturation at approximately 180 °C toward 1% of hydrogen gas (Figure 30a). The authors also reported a severe decrease in the working temperature of Pd/SnO_2_ sensor response to 1% of H_2_ gas below 50 °C (Figure 30b). Moreover, pure SnO_2_ and Pd/SnO_2_ demonstrated reasonable sensitivity even toward 50 ppm of target gas (Figure 30c) at 180 °C and 50 °C, respectively. A comparative response of pure SnO_2_ and Pd/SnO_2_ nanospheres showed that both synthesized sensors’ response and recovery time at their corresponding operating temperature was less than 10 s and about 20 s, respectively (Figure 30d). To evaluate the selectivity of Pd/SnO_2_ The responses of Pd/SnO_2_ sensors towards ethanol, acetone and ammonia at different temperatures were explored. At higher temperature, the sensitivity of sensor to the test gases increased. However, it shows a higher selectivity for the detection of hydrogen at lower temperature (below 60 °C) within an interval of 10 s.

Another study by Gattu et al. reported the gas-sensing characteristics of green synthesized un-doped and Fe-doped SnO_2_ nanoparticles [225] from remnant water (kitchen waste) of soaked Bengal gram beans (*Cicer arietinum*). This extract contains different bio-molecules that act as complexing and capping agents to synthesize Fe-doped SnO_2_ nanoparticles. The sensor response of pure SnO_2_ and Fe-doped SnO_2_ thin film to 100 ppm of NH_3_ gas was 28% and 46%, respectively, at the operating temperature of 200 °C. Additionally, the Fe-doped SnO_2_-based sensor showed higher selectivity to NH_3_ gas at 200 °C in comparison with the un-doped tin oxide-based sensor.

A summary of the SnO_2_-based gas sensor is reported in Table 10.

**Table 10:** A comparison of gas sensing characteristics of SnO_2_-based gas sensor.

Material	Structure/Synthesis Method	Target Gas/Concentration (ppm)	Operating T (°C)	Response (%)	Ref.
SnO_2_	-/green Pechini	Ethanol/50	276	27	[226]
Ce–SnO_2_			265	69.5	
SnO_2_	spherical/ hydrothermal	Ethanol/100	230	24.9	[227]
Carbonous particles/SnO_2_	hollow nanosphere/-	Ethanol/10	200	7	[221]
		Ethanol/20		13	
		Ethanol/30		22	
		Ethanol/40		39	
		Ethanol/150		93	
		Ethanol/200		127	
		Ethanol/250		134	
SnO_2_	thin-film/-	NO_2_/20, 60, 100	200	28	[223]
Ni-doped SnO_2_		NO_2_/20, 60, 100		40	

### 6.9. Tungsten (Tri) Oxide Nanoparticles (WO_3_ NPs)

Tungsten oxide behaves as an n-type transition metal oxide semiconductor with a bandgap ranging from 2.5 to 3.6 eV. It possesses several exciting characteristics like excellent stability (over a wider temperature range), unique electrical and optical features, low cost, non-toxicity, and good gas sensitivity to oxidizing and reducing gases [228,229]. These advantages make it a promising candidate in a variety of applications such as photocatalysis, antimicrobial activity against human pathogens [230], electrochromic display, chemical sensing [231,232], biosensing [233] and gas sensing. Tungsten oxide has drawn significant interest as sensor materials [234] such as resistive [235], optical [236], and capacitive [237] devices for a large variety of different gas analytes such as NO_x_, NH_3_, CO, H_2_, O_3_, ethanol, and H_2_S [234]. The gas sensing mechanism of WO_3_ is attributed to the surface-controlled type [238].

**Figure 31:**
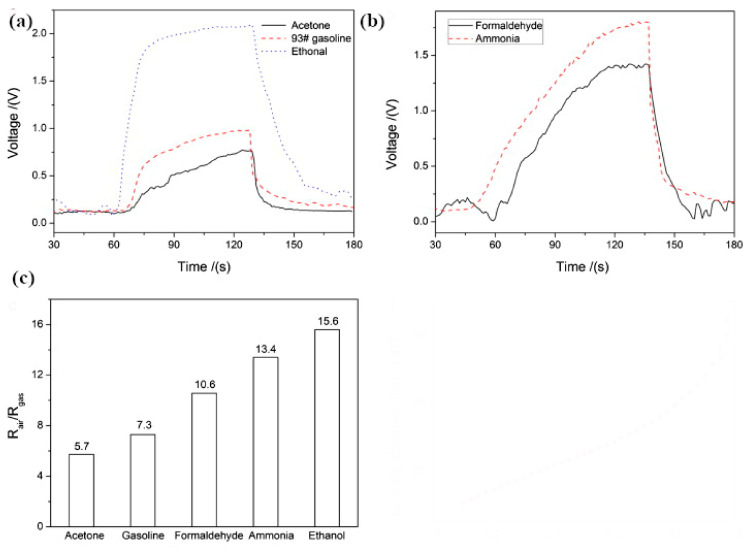
Sensor response of WO_3_ monohydrate nanosheets: (**a**,**b**) Response curves of the sensor to flammable organic vapors and pure gases; (**c**) Sensor sensitivity under various gases. Reprinted from Ref. [238] with permission of Materials Chemistry and Physics, 2011.

Researchers have synthesized WO_3_ nanomaterials with various dimensions such as nanoparticles (0D) [141], nanofibers (1D) [142], nanosheets (2D) [143], nanosphere [144], nanoflowers [239], and nanomesh (3D) [145]. There are several methods to synthesize tungsten oxide, including acid precipitation, wet chemical method, sol-gel, ion-exchange approach with sodium tungstate as a precursor, etc. [238]. Nevertheless, traditional methods suffer many limitations and challenges like the utilization of harmful and expensive chemicals, large-scale production of the nanomaterials having improved features than their commonly available bulk form, and their practical application [238,240]. Hence, the biosynthesis of tungsten oxide has gained increased consideration to decrease the use of toxic chemicals. Kavitha et al. [126] synthesized tungsten oxide nanoflakes (WO_3_ NFs) using the plant pathogenic fungus *F. solani* with an average thickness and length of around 40 nm and 300 nm, respectively. Tijani et al. [128] produced WO_3_ nanoparticles using *Spondias mombin* aqueous extract, and they examined the effect of solution pH (1, 4, 7, 10, 13) and calcination temperature (250 °C, 350 °C, 450 °C, 550 °C, 650 °C, each for 2 h) on the morphological properties, crystallite size and surface area of the nanoparticles. The average crystallite size was found to be 13.1, 14.7, 25.2, 27.1, 29, and 31 nm for WO_3_ nanoparticles prepared at pH = 1 and calcined at 250°, 350°, 450°, 550°, and 650 °C, respectively. The SSA of WO_3_ NPs prepared at pH 1 was higher than other pH values, probably because of a higher rate of electron transfer in an acidic medium than in a neutral or basic medium. The SSA increased from 156.3 m^2^/g up to 352.6 m^2^/g when the firing temperature was increased from 250 to 550 °C and then, was reduced to 18.1 m^2^/g when the treatment was operated at 650 °C. Spherical tungsten nanoparticles with an average size of 10 nm were synthesized through the green method using aqueous extracts of *Moringa oleifera* [127] to test different biological activities. Orthorhombic tungsten trioxide monohydrate nanosheets (WONSs) in high yields were successfully green synthesized by Chang et al. [238] and used as a gas sensing material. The prepared sensors were then tested at room temperature in presence of three different flammable organic vapors (Figure 31a) and two harmful gases (Figure 31b). The response curves towards 100 ppm ethanol, acetone, and 93# gasoline are shown in Figure 31a. The experimental results showed that the output voltage dramatically increased when the sensor was exposed to ethanol vapor, indicating a decrease in the sensors’ electrical resistance. The WONSs sensor showed a short response (~15 s) and recovery (~28 s) times toward 100 ppm ethanol.

Furthermore, biosynthesized sensors exhibited high sensitivity of 15.6 under 100 ppm of ethanol (Figure 31c). However, the sensor possessed relatively weaker responses to gasoline and acetone vapors with 7.3 and 5.7, respectively (Figure 31c), respect to ethanol, even though the WONSs sensor had fast response and recovery times to acetone. Besides these, the WONSs sensors had an excellent sensor response toward ammonia and formaldehyde with 13.4 and 10.6, respectively, which were lower than the one under ethanol vapor (Figure 31b). Nevertheless, they reported longer response times to both detected gases, whereas they had short recovery times.

## 7. Future Perspectives

There are several significant ways to maximize the performance of “green” chemistry in all aspects of life [5,42] by means of the following:

- Utilization of innovative alternative routes to minimize the harmful effects on the human health and the environment such as improvement of catalytic efficiency, production of less toxic waste, use of less hazardous solvents such as ionic liquids, water, etc. instead of dangerous reagents, use of renewable sources of precursors and least consumption of energy.
- Higher diffusion within the community of researchers studying gas sensors of green synthesis principles as in other fields of science.
- Organization of different chemical associations and institutes to study cleaner and safer reactions and methods with cleaner productions.
- Promotion of green synthesis at universities and research laboratories for the development of this approach in terms of economy.
- Introduction of green chemistry to industrial enterprises.
- Training the researchers who will solve the environmental problems.
- Enhancement of environmental protection at the legislative level.
- Assessment of toxicity nature and effective risk management associated with nanoparticles, their synthesis, handling, and storage.
- In the future, biological methods for the synthesis of nanomaterials may focus on the reduction of manufacturing time.

Scientific investigations of green synthesis methods should be considered and used in various industrial products, including not only gas sensors but also energy, medicines, cleaning products, food industry, plastics, cosmetics, etc.

## 8. Conclusions

Recently, air pollution has become a severe threat to humans and the ecosystem. Therefore, it is essential to detect air pollutants, such as NO_2_, NO, N_2_O, H_2_S, CO, NH_3_, CH_4_, SO_2_, CO_2_, and BTX (Benzene, Toluene and Xylene), etc., beyond their tolerable limits.

Bottom-up methods are commonly used to fabricate metal/metal oxide nanoparticles by applying different organic solvents and toxic and non-ecological reagents under high pressure and temperature. For this reason, the development of alternative cheap and safe techniques is essential to reduce negative effects on human health and the environment.

Various biological routes have been developed using “green” resources like plants, algae, fungi, yeast, bacteria, viruses, etc. Among these biosources, plants are the best source as the raw materials to synthesize metal/metal oxide nanoparticles. They are known for their non-toxicity, availability, simplicity, antioxidant capacity, and protein content. Different plant extracts can be used as reducing, capping, and stabilizing agents. Although there is a limited but essential role of green chemicals in nanoparticle synthesis, it has to be underlined that there is a considerable availability of plant extracts worldwide that can act as reducing, capping, and stabilizing agents. Thus, their use, not so diffused for MOS synthesis, should be greatly encouraged. In the separate section of this review paper, the biological synthesis and the use of metal oxide nanoparticles such as ZnO, TiO_2_, CeO_2_, SnO_2_, In_2_O_3_, CuO, NiO, WO_3_, and Fe_3_O_4_ for gas sensing applications are reported. The obtained gas sensors received widespread attention due to their particular characteristics.

The set-up of high-performance gas sensors is a hot topic that has attracted considerable attention from researchers involved in their development over several decades. Specifically, huge efforts have been made to improve the sensor response of gas sensors by doping them with noble metals (to favor oxygen molecules adsorption and spillover) and other metal oxides (to form hetero-junctions) as well as by tailoring the microstructures (targeting hierarchical, hollow and porous structures) with the aim to increase the SSA and to shorten response/recovery times. Thus, important results have already been reached and these strategies are still under investigation. However, facet-controlled synthesis is also one very promising strategy to enhance sensing performances, because special facets with high surface energy, dangling bonds and densities of atoms also present a much higher chemical activity. In fact, using the “green” approach to synthesizing metal and metal oxide nanoparticles allows for obtaining nanoparticles with desirable size and improved morphology. Thus, this technology can not only decrease the risk to human health and environmental pollution but can also help to produce sensors with the right microstructure that present the highest response to the target gas.

## Figures and Tables

**Table 1:** Advantages and disadvantages of the green synthesis method. Elaboration from Ref. [43].

Green Synthesis Method	
Merits	Drawbacks
Environmentally friendly process Controlled shape and size of NPs Without contamination compared to the traditional process Prevention of wastes/by-products Decrease derivative products Without the requirement of high energy, temperature, and pressure Renewable materials are easily accessible Large scale production of NPs Degradable chemical products Cost-effective and easy implementation In-process monitoring Reduced cases of accidents	Limited applications in the industry Increase risks of NPs entering the body leading to inhalation problems and lethal diseases Limited studies have been conducted on the bioaccumulation of NPs and their toxicity in the environment

**Table 2:** Applications of green synthesized metal and metal oxide nanoparticles. Elaboration from refs. [42,44].

Metal/Metal Oxide Nanoparticles	Applications
Silver (Ag), Gold (Au), Platinum (Pt), Palladium (Pd), Copper (Cu)	Antimicrobial, Fuel cells, Catalysts, Sensor, Drug delivery
Aluminum oxide (Al_2_O_3_)	Antimicrobial, Sensor
Cerium oxide (CeO_2_)	Antimicrobial, Biomedical, Drug delivery, Anticancer
Titanium oxide (TiO_2_)	Catalyst, Sensor, Electronic, Anticancer, Antifungal, Cosmetics
Copper oxide (CuO)	Antimicrobial, Sensor, Drug delivery
Zinc oxide (ZnO)	Catalyst, Antimicrobial, Sensor, Drug delivery
Nickel oxide (NiO)	Biomedical, Sensor, Photocatalytic, Antibacterial, Antifungal
Iron oxide (Fe_3_O_4_)	Antimicrobial, Sensor, Drug delivery

**Table 3:** The gas-sensing performance of n-type and p-type MOS towards reducing and oxidizing gases.

Type and Examples of MOS	Reducing Gases (H_2_, CO, CH_4_, Ethanol, and Acetone)	Oxidizing Gases (O_2_, O_3_, NO_x_, SO_2_)	Dominant Charge Carrier	Response
n-type (ZnO, SnO_2_, TiO_2_, WO_3_, In_2_O_3_, etc.)	Decreasing the resistance	Increasing the resistance	Electrons	S^n^ = R_g_/R_a_ (Oxidizing gases) S^p^ = R_a_/R_g_ (Reducing gases)
p-type (Cu_2_O, Co_3_O_4_, Cr_2_O_3_, Mn_3_O_4_, NiO, etc.)	Increasing the resistance	Decreasing the resistance	Holes	S^n^ = R_g_/R_a_ (Reducing gases) S^p^ = R_a_/R_g_ (Oxidizing gases)

S: Sensor response; R_a_: Sensor resistance in air; R_g_: Sensor resistance in the mixture of target gas and air.

**Table 4:** Synthesis of metal oxide nanoparticles (MO NPs) from various bio species.

Plants Name/Part	MO NPs	Shape Of NPs	Size of NPs (nm)	Ref.
*Persia americana*/seed	SnO_2_	Flake-like	4	[88]
*Annona squamosa*/peel		Spherical	27.5	[89]
*Aspalathus linearis*/leaf		Quasi-spherical	2.5–11.40	[90]
*Catunaregam spinosa*/root		Spherical	47	[91]
*Eupatorium odoratum (Chromolaena odorata*)/leaf	CuO	Spherical	-	[92]
*Hylotelephium telephium*/ flower		Spherical	83	[93]
*Coriandrum sativum* L./seed		Irregular	18.2	[94]
*Punica granatum*/fruits peel		Spherical	10–100	[95]
*Bifurcaria bifurcata*/Alga		Spherical	5–45	[96]
*Olea europaea*/ leaf		Nanobullets	-	[97]
*Hibiscus sabdariffa*	CeO_2_	Spherical	3.9	[98]
*Aloe vera*/leaf		Spherical	2–3	[99]
China rose/flower petal		Nanosheet	7	[100]
*Curvularia lunata*/fungus		Spherical	5–20	[101]
Watermelon/fruit		-	36	[102]
*Kappaphycus alvarezii*/seaweed	Fe_3_O_4_	Spherical to agglomerate	11–20	[103]
*Carica papaya*/leaf		Spherical to agglomerate	33	[104]
Plantain/peel		Spherical	<25	[105]
*Syzygium cumini*/seed		Spherical to agglomerate	~14	[106]
*Sargassum muticum*/Alga		Cubic	18 ± 4	[107]
*Catharanthus roseus*/leaf	TiO_2_	Clustered and irregular	25–110	[108]
*Mangifera indica*/leaf		Spherical, Oval to agglomerate	30 ± 5	[109]
*Bacillus amyloliquefaciens*/bacterium		Spherical	15.23–87.6	[110]
*Annona squamosa*/fruit peel		Spherical	23 ± 2	[111]
*Aeromonas hydrophila*/bacterium		Spherical	28–54	[112]
*Nyctanthes arbortristis*/flower	ZnO	Agglomerates	12–32	[113]
*Beta vulgaris*/root		Agglomerates	~52–76	[114]
*Sargassum muticum*/Alga		Hexagonal	3–57	[115]
*Moringa oleifera*/leaf		Spherical	~6–10	[116]
*Carissa edulis*/fruit		Agglomerates	50–55	[117]
*Vernonia amygdalina*/ leaf	NiO	Octahedral	-	[118]
*Raphanus sativus*/root		-	34.89	[119]
*Limonia acidissima*/ fruit		Spherical	~23	[120]
*Nigella sativa*/Seed		Spherical, agglomerated	10–50	[121]
*Microbacterium* sp. MRS-1/Bacteria		Flakes	100–500	[122]
*Hypocrea lixii*/Fungi		Spherical	1.25–3.8	[123]
*Rhodotorula mucilaginosa*/Yeast		Spherical	5.5	[124]
Red marine algae/Algae		Uniform, smooth and non-spherical	32.64	[125]
*Fusarium solani*/Fungus	WO_3_	Nanoflakes	Thickness (40) length (~300)	[126]
*Moringa oleifera*/aqueous extracts		Spherical	10	[127]
*Spondias mombin*/leaf		Spherical (pH = 1)	10–13	[128]
		Purely hexagonal (pH = 4)	12–14	
		Hexagonal–spherical (pH = 7)	13–16.7	

**Table 5:** Metal oxides for application in gas sensors. Elaboration from Refs. [19,79,131].

Target Gas	Metal Oxide Composites and Mixed Metal Oxides
CO	SnO_2_-Co_3_O_4_; SnO_2_-Fe_2_O_3_; SnO_2_-Mn_2_O_3_; In_2_O_3_-SnO_2_; In_2_O_3_-Ga_2_O_3_; In_2_O_3_-Co_3_O_4_; (Pt, Pd, Au)-loaded WO_3_
H_2_	In_2_O_3_-SnO_2_; SnO_2_-CuO; SnO_2_-ZnO; SnO_2_-TiO_2_; TiO_2_-NiO; In_2_O_3_-ZnO
NH_3_	SnO_2_-MoO_3_; SnO_2_-Fe_2_O_3_; ZnO-CuO; ZnO-MnO_2_; α-Fe_2_O_3_-ZnO; TiO_2_-CuO; TiO_2_-Cr_2_O_3_; h-WO_3_
NO_x_	ZnO-SnO_2_; ZnO-CdO; ZnO-Al_2_O_3_; In_2_O_3_-ZnO; In_2_O_3_-SnO_2_; Fe_2_O_3_-SnO_2_; SnO_2_-NiO; WO_3_; WO_3_-TiO_2_
Ethanol	α-Fe_2_O_3_-SnO_2_; α-Fe_2_O_3_-ZrO_2_; α-Fe_2_O_3_-TiO_2_; α-Fe_2_O_3_-In_2_O_3_; α-Fe_2_O_3_-CuO; In_2_O_3_-ZnO; In_2_O_3_-SnO_2_; SnO_2_-CuO; SnO_2_–TiO_2_; SnO_2_-NiO; SnO_2_-ZnO; Co_3_O_4_-ZnO; TiO_2_-CuO
VOCs	SnO_2_-ZnO; SnO_2_-In_2_O_3_; SnO_2_-CuO; SnO_2_-NiO; Fe_2_O_3_-SnO_2_; α-Fe_2_O_3_-NiO; Co_3_O_4_-ZnO; ZnO-CuO
CH_4_	In_2_O_3_-SnO_2_; In_2_O_3_-SnO_2_-TiO_2_; SnO_2_-CaO; WO_3_
O_3_	In_2_O_3_-Fe_2_O_3_; In_2_O_3_-NiO; In_2_O_3_-ZnO-SnO_2_; In_2_O_3_-SnO_2_
H_2_S	SnO_2_-CuO; SnO_2_-Ag_2_O; ZnO-CuO; WO_3_-CuO; Fe_2_O_3_-SnO_2_; CdO–In_2_O_3_; (Pt, Au, Au–Pt)-loaded WO_3_
CO_2_	BaTiO_3_-CuO-La_2_O_3_; CuO-CuxFe_3−x_O_4_; SnO_2_-La_2_O_3_; ZnO; WO_3_-TiO_2_
SO_2_	TiO_2_-V_2_O_5_; V_2_O_5_-WO_3_-TiO_2_; SnO_2_-NiO; WO_3_; Pt-WO_3_
Cl_2_	NiFe_2_O_4_; ZnFe_2_O_4_; In_2_O_3_-Fe_2_O_3_

**Table 6:** A brief summary of green-synthesized zinc-oxide as gas sensors.

Material	Structure/Synthesis Method	Target Gas/Concentration (ppm)	Operating T (°C)	Response (%)	Ref.
ZnO-CAL (YHLP)	Spherical and hexagonal	LPG	100	14	[86]
			200	22.5	
			230	31.05	
			300	21.9	
			400	11	
			500	5.8	
ZnO	film/drop-casting	H_2_/0.5%	150	24	[158]
		H_2_/2%		46	
ZnO	film/precipitation	Ethanol vapor/40	-	5.4	[160]
		Ethanol vapor/80		5.56	
		Ethanol vapor/120		6.22	
		Ethanol vapor/160		9.94	
		Ethanol vapor/400		13.63	
		Ethanol vapor/800		28.7	
ZnO	thin film/-	NH_3_/600	150	~57	[167]
ZnO@ZIF-8	Core-Shell /DGC	H_2_/50	-	0.9	[168]
		H_2_/100		2.4	
ZnO	hexagonal/-	NH_3_/0.5	80	~1.07	[161]
		NH_3_/1		~1.2	
		NH_3_/2		~1.5	
		NH_3_/5		~2.5	
		NH_3_/10		~3.0	

YHLP: *Young Harbara* Leaves Plant; LPG, Liquefied petroleum gas; DGC: Dry Gel Conversion.

**Table 7:** Iron oxide nanoparticles were obtained using the green synthesis method.

Material	Structure/Synthesis Method	Target Gas/Concentration (ppm)	Operating T (°C)	Response (%)	Ref.
γ-Fe_2_O_3_	Chains/-	Methane/100	50	2	[104]
			70	7	
			90	12	
			110	15	
			130	20	
			150	36	
			170	32	
			190	29	
γ-Fe_2_O_3_	Chains/-	Methane/1	150	15	
FeYO_3_	Hexagonal perovskite/hydrothermal	Ethanol/1	330	2.38	[194]
		Ethanol/25		-	

**Table 8:** A summary of green synthesized In_2_O_3_ nanoparticles as a gas sensor.

Material	Structure/Synthesis Method	Target Gas/Concentration (ppm)	Operating T (°C)	Response (%)	Ref.
In_2_O_3_	Hollow sphere/hydrothermal	alcohol, acetone, gasoline, formaldehyde, chloroform, and acetonitrile	-	-	[199]
In_2_O_3_	cubes/hydrothermal	NO_2_/5	100	1.3	[197]
		NO_2_/10		9	
		NO_2_/20		20	
		NO_2_/40		80	
		NO_2_/60		140	
		NO_2_/80		350	
		NO_2_/100		600	
		CO_2_/100		18	
		NH_3_/100		8	
		Acetone/100		7	

**Table 9:** A comparison of various synthesis methods of copper oxide nanoparticles. Elaboration from ref. [206].

Synthesis Route	Merits	Demerits
Physical	Controlled shape, size, and crystallinity High uniformity and purity	Needs of high energy
Chemical	A large scale of production	Use of toxic solvent Non-eco-friendly products High energy consumption
Biological	Cost-effective, facile and eco-friendly	The use of microorganisms is not desirable
